# Insight into small molecule binding to the neonatal Fc receptor by X-ray crystallography and 100 kHz magic-angle-spinning NMR

**DOI:** 10.1371/journal.pbio.2006192

**Published:** 2018-05-21

**Authors:** Daniel Stöppler, Alex Macpherson, Susanne Smith-Penzel, Nicolas Basse, Fabien Lecomte, Hervé Deboves, Richard D. Taylor, Tim Norman, John Porter, Lorna C. Waters, Marta Westwood, Ben Cossins, Katharine Cain, James White, Robert Griffin, Christine Prosser, Sebastian Kelm, Amy H. Sullivan, David Fox, Mark D. Carr, Alistair Henry, Richard Taylor, Beat H. Meier, Hartmut Oschkinat, Alastair D. Lawson

**Affiliations:** 1 Leibniz-Forschungsinstitut für Molekulare Pharmakologie, Berlin, Germany; 2 UCB Celltech, Slough, United Kingdom; 3 Laboratory of Physical Chemistry, ETH Zürich, Zürich, Switzerland; 4 Sanofi, Strasbourg, France; 5 Evotec, Milton, United Kingdom; 6 Midatech Pharma Plc, Milton, United Kingdom; 7 Leicester Institute of Structural and Chemical Biology, University of Leicester, Leicester, United Kingdom; 8 Vertex, Milton, United Kingdom; 9 Beryllium Discovery, Bedford, Massachusetts, United States of America; UMDNJ/Robert Wood Johnson Medical School, United States of America

## Abstract

Aiming at the design of an allosteric modulator of the neonatal Fc receptor (FcRn)–Immunoglobulin G (IgG) interaction, we developed a new methodology including NMR fragment screening, X-ray crystallography, and magic-angle-spinning (MAS) NMR at 100 kHz after sedimentation, exploiting very fast spinning of the nondeuterated soluble 42 kDa receptor construct to obtain resolved proton-detected 2D and 3D NMR spectra. FcRn plays a crucial role in regulation of IgG and serum albumin catabolism. It is a clinically validated drug target for the treatment of autoimmune diseases caused by pathogenic antibodies via the inhibition of its interaction with IgG. We herein present the discovery of a small molecule that binds into a conserved cavity of the heterodimeric, extracellular domain composed of an α-chain and β2-microglobulin (β2m) (FcRn_ECD_, 373 residues). X-ray crystallography was used alongside NMR at 100 kHz MAS with sedimented soluble protein to explore possibilities for refining the compound as an allosteric modulator. Proton-detected MAS NMR experiments on fully protonated [^13^C,^15^N]-labeled FcRn_ECD_ yielded ligand-induced chemical-shift perturbations (CSPs) for residues in the binding pocket and allosteric changes close to the interface of the two receptor heterodimers present in the asymmetric unit as well as potentially in the albumin interaction site. X-ray structures with and without ligand suggest the need for an optimized ligand to displace the α-chain with respect to β2m, both of which participate in the FcRn_ECD_–IgG interaction site. Our investigation establishes a method to characterize structurally small molecule binding to nondeuterated large proteins by NMR, even in their glycosylated form, which may prove highly valuable for structure-based drug discovery campaigns.

## Author summary

In drug design, a detailed characterization of structural changes induced by drug binding is useful for further optimizing lead compounds. In many cases, structural alterations are distant from the compound binding site, potentially acting through allosteric effects. These allosteric effects are often difficult to observe by static methods, i.e., X-ray crystallography, but can be monitored by NMR spectroscopy. The latter method, however, has size-limitations when investigating the protein backbone structure in solution-state. To overcome this, we present an innovative approach employing ultrafast magic-angle-spinning (MAS) NMR on the extracellular domain of the neonatal Fc receptor (FcRn_ECD_). This is a validated drug target in autoimmune diseases, and we aim to identify and characterize novel compounds to serve as starting points to develop allosteric inhibitors of this receptor. After sedimentation, we could record well-resolved proton-detected MAS NMR spectra of the fully protonated [^13^C,^15^N]-labeled protein, enabling the observation of structural changes. In combination with computational methods, X-ray crystallography, and other biophysical tools, we present new compounds that may be used as allosteric modulators of FcRn after further optimization. The introduced MAS NMR approach can be applied to a large variety of proteins to support structure-based drug design, facilitating the detection of allosteric effects.

## Introduction

In order to discover new chemical drugs, fragment screening followed by structure-based design is an efficient way to sample chemical space and find hits for challenging target classes such as protein-protein interactions [1–3]. In addition to discovering orthosteric ligands, fragment screening has the potential to locate secondary binding sites on a protein that may be exploited for allosteric regulation [4]. In the development process, a methodology that includes detection of allosteric effects is highly welcome. Magic-angle-spinning (MAS) NMR has the potential to contribute via the detection of long-range chemical-shift changes when the investigated protein is too large for solution-state NMR and can even not be deuterated. It is applied here to a soluble 42 kDa construct of the neonatal Fc receptor (FcRn) within a search for allosteric regulators, employing very fast MAS (100 kHz).

**Fig 1:**
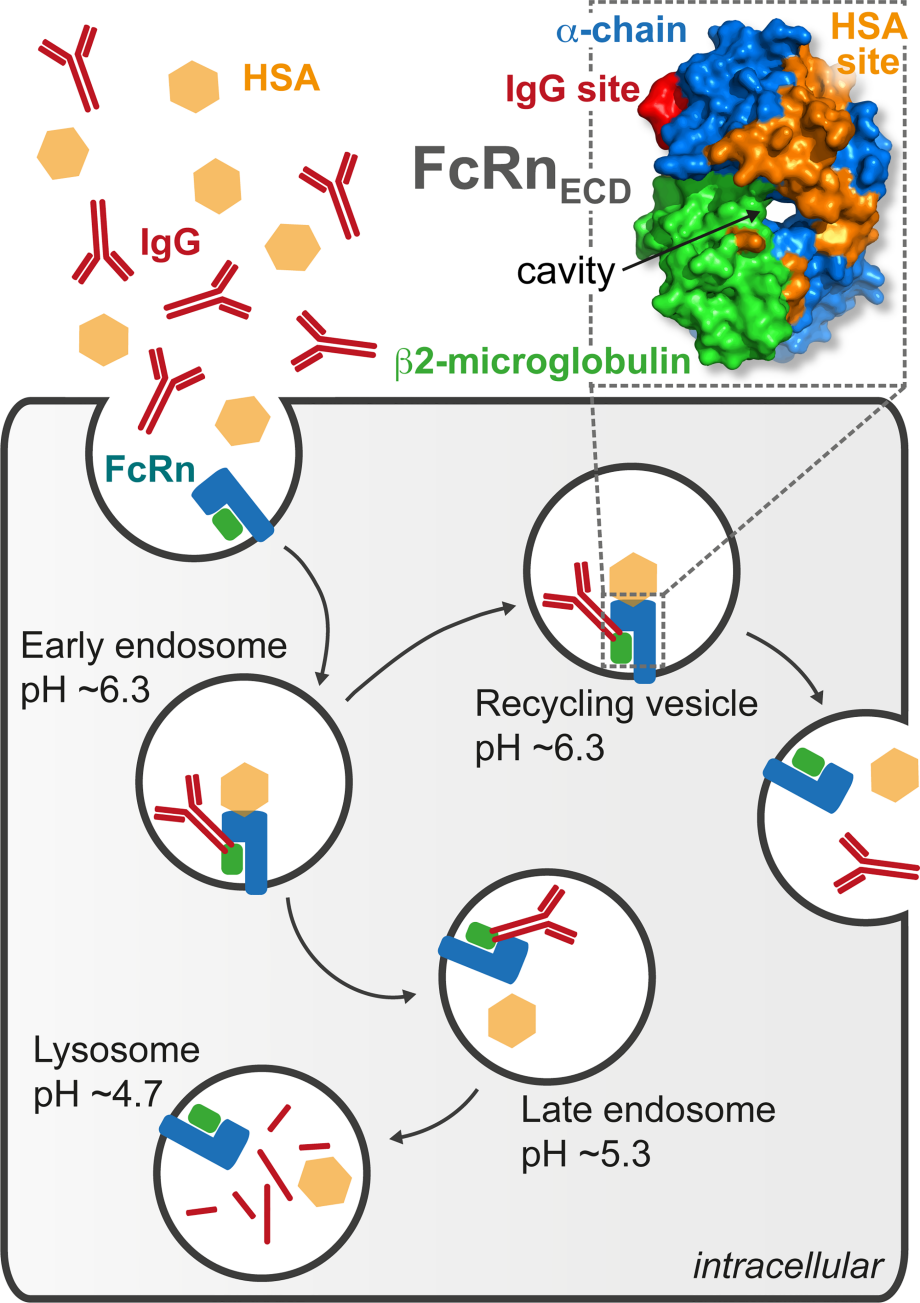
FcRn allows maintenance of protein homeostasis. The soluble extracellular domain of neonatal Fc receptor (FcRn_ECD_, PDB code 1EXU) is a heterodimer composed of β2m (green) and α-chain (blue) with a cavity at the interface between the two proteins. FcRn is involved in the regulation of HSA (orange) and IgG (red) levels. The binding of both HSA and IgG to FcRn is pH dependent, which provides a mechanism for protein homeostasis through endosomal trafficking. β2m, β2-microglobulin; FcRn, neonatal Fc receptor; FcRn_ECD_, extracellular domain of the neonatal Fc receptor; HSA, Human Serum Albumin; IgG, Immunoglobulin G; PDB, Protein Data Bank.

FcRn facilitates new-born humoral immunity by regulating Immunoglobulin (IgG) transport across the epithelium [5]. In addition, it has been shown to bind to IgG and Human Serum Albumin (HSA) at nonoverlapping sites in a pH-dependent manner (Fig 1) [6,7]. This allows maintenance of IgG and HSA homeostasis, accounting for the long serum half-life of both proteins [8–11]. At low pH, the interaction of FcRn with IgG occurs through protonation of ionizable residues, located at the CH2–CH3 hinge of the IgG Fc, which produces transient, intermolecular salt bridges with negatively charged residues on FcRn [12]. The interaction of FcRn with IgG and HSA occurs in acidified early endosomes, diverting the proteins from catabolism and carrying them back to the neutral pH environment of the extracellular compartment. At near-neutral pH, the affinity of the interaction decreases, and the complex dissociates [10,13].

Existing as a heterodimer composed of β2-microglobulin (β2m) and a membrane-anchored α-chain (Fig 1), FcRn is homologous to the class I major histocompatibility complex (MHC1) [14]. MHC1 presents antigenic peptide fragments to the T cell receptor (TCR) in complex with cluster of differentiation 8 (CD8) [14]. In contrast, FcRn is not involved in endogenous peptide presentation to the TCR; its peptide-binding groove is closed and nonfunctional [14].

The effect of FcRn loss has been studied using β2m-deficient mice, which develop normally but are defective in T cell–mediated cytotoxicity [15]. Deletion of β2m precluded FcRn expression, resulting in a reduction in IgG half-life [16–18]. Additionally, random mutation of residues proximal to the IgG–FcRn binding site allowed selection of Fc variants with increased affinity for FcRn at pH 6. These Fc constructs maintained pH-dependent binding and showed an extended half-life relative to wild-type IgG [19].

FcRn has been proposed as a drug target in the treatment of autoimmune diseases, in which pathogenic autoantibodies are detrimental to health [20]. Examples of such diseases include, but are not restricted to, myasthenia gravis, Guillain–Barré syndrome, and dermatomyositis [21–24]. Antibodies produced against the FcRn heavy chain ameliorated myasthenia gravis symptoms in rats, and it has been shown that mice without FcRn are resistant to autoimmune disease [25–27]. The current treatment for these conditions is intravenous application of immunoglobulin, which increases the turnover of pathogenic IgG by saturating FcRn [26]. Recently, rozanolixizumab, an antihuman FcRn monoclonal antibody, reduced the serum IgG concentration in a randomized phase 1 study, providing clinical evidence for the potential of an anti-FcRn therapeutic [28].

A chemical inhibitor to control FcRn trafficking is therapeutically desirable for potential treatment of autoimmune disorders. Orthosteric peptide inhibitors with in vivo efficacy have previously been reported [29–33]. Additionally, structure–activity relationships of small molecule antagonists using ELISA assays have also been described [34]. However, to date, no allosteric modulators of FcRn–IgG or FcRn–HSA interactions have been reported. Should such molecules be found, they could be used therapeutically or as tools in biomedical investigations.

In this study, we present the discovery of a compound that binds to the soluble extracellular domain of FcRn, abbreviated here as FcRn_ECD_. In the initial screening process, complementary in silico methods were used to predict binding sites on the basis of charge and topography or sequence conservation, alone and in combination [35–37]. The interaction of the ligand with FcRn_ECD_ is investigated by a combination of X-ray crystallography and proton-detected, ultrafast MAS NMR, revealing its localization in an evolutionarily conserved binding pocket. At high MAS frequencies of 100 kHz, we could acquire well-resolved proton-detected NMR spectra on sedimented, fully protonated FcRn_ECD_, allowing de-novo chemical-shift assignments of residues in the binding pocket and in β2m through establishing sequential connections. MAS NMR pinpoints chemical-shift changes upon ligand binding close to the binding site and in regions distant to it. In this context, MAS NMR helps to exclude the influence of crystal packing effects by making use of protein solutions. Both crystal structures with and without ligand were available, but structural changes were not obvious when using global fitting procedures, with the molecules possibly “locked” into a conformation by crystal contacts. To identify sections of the structure not affected by ligand binding for a fit that is also sensitive to small allosteric effects, residues that did not show chemical-shift changes were used to produce “chemical-shift–informed” overlays of FcRn_ECD_ X-ray structures with and without ligand. Our findings suggest that therapeutic intervention in autoimmune diseases may be achieved through allosteric small molecules that bind to FcRn_ECD_. In addition, such compounds could potentially be used as chemical probes to study FcRn trafficking. The presented approach highlights further the use of MAS NMR for detecting structural changes in nondeuterated proteins expressed in mammalian cells upon ligand binding.

## Results and discussion

### X-ray crystallography of FcRn_ECD_ and ligandability assessments

Small molecule binding sites on proteins can be identified based on surface shape, charge, and functionality. We used SiteMap software to identify binding sites on the FcRn_ECD_ [35]. To obtain structural data for our analysis, diffraction data for FcRn_ECD_ crystals, at pH 3 and pH 8.5, were collected at cryogenic temperatures and structures solved to 2.0 Å and 2.45 Å, respectively, by molecular replacement. Two copies of FcRn_ECD_ were found in the asymmetric unit. In addition to the structures at acidic and basic pH, a pH 7.2 structure was generated using molecular dynamics simulation (S1 Fig and S1 Text).

The SiteMap software detected a number of regions with a druggability score >1.0, a nominal quantifier indicating that nM binding might be achieved with a conventional small molecule of <500 Da molecular weight. In particular, SiteMap identified a ligandable site at the interface between the α-chain and β2m (S1 Fig and S1 Text). The respective boundaries were predicted to vary with pH, with the area described as either one large or three distinct cavities.

No regions near the IgG binding site were found by SiteMap. Based on our analysis, should an orthosteric pocket for an IgG blocking small molecule exist, it is likely to be transient in nature and not stabilized in the crystal lattice.

### Evolutionary conservation of FcRn_ECD_

On the premise that evolutionarily conserved cavities may have an associated function, we evaluated such conservation of the cavities found by SiteMap (S2 Fig). We identified ortholog sequences using OrthoDB and performed sequence alignments using Clustal Omega [38,39]. A homology model was then created for each sequence using MEDELLER that enabled us to visualize mutations according to the evolutionary conservation in PyMOL [40,41].

Our sequence analysis broadly supports the notion that regions of the protein important for structural integrity or function are conserved, in particular the interface between the α-chain and β2m. It contains key contacts, such as those between D53_β2m_, Q34_α-chain_, and S37_α-chain_, and conservation of these contacts preserves the structural integrity of the noncovalently linked heterodimer.

Additionally, motifs within the HSA binding site were also conserved. One of them has previously been identified as being critical for albumin binding and was the site of a protein contact in our pH 3 and pH 8.5 crystal structures, between the two copies of FcRn_ECD_ in the asymmetric unit [8]. We were initially surprised to see the IgG binding site is poorly conserved with the exception of key residues, such as D130_α-chain_. This may be attributed to the heterogeneity in Fc moieties between mammalian orthologues, which presumably reduces species’ cross-reactivity. Of the regions identified by SiteMap, the central cavity was highly conserved, potentially due to the proximity to the dimer interface.

### Fragment screening to identify small molecules

Our in silico analysis suggests the presence of an evolutionarily conserved binding site at the interface between the α-chain and β2m. To find ligands for this cavity and, potentially, other sites, a solution-state NMR fragment screen was performed. For this purpose, we used an in-house library of approximately 1,100 molecules containing fluorine atoms to enable ligand interrogation by ^19^F Carr-Purcell-Meiboom-Gill NMR [42,43]. This screen detected 143 potential binders. In order to select compounds for crystallographic studies, active molecules were further tested in Saturated Transfer Difference NMR experiments and by Surface Plasmon Resonance (SPR), yielding an estimated K_D_ of 80 μM for the highest affinity fragment, the racemate UCB-FcRn-84 (S3 Fig and S1 Data) [44,45]. Chiral separation showed preferential binding of the R enantiomer. Competition of UCB-FcRn-84 with IgG was tested in a FcRn_ECD_–IgG FRET assay; however, no measurable inhibition was observed.

### Crystallographic studies of ligand-bound FcRn_ECD_

Confirmed hits from the fragment-screen were soaked into FcRn_ECD_ crystals that had been grown under acidic conditions. Based on the derived crystal structures, we aimed to further improve both affinity and solubility of the ligand.

UCB-FcRn-84 was found in the conserved cavity at the interface of β2m and the α-chain (S4 Fig and S2 Text), as predicted by SiteMap. Key properties of binding interactions include burial of the 3-fluorophenyl group in a hydrophobic cavity composed of Y26_β2m_, S52_β2m_, Y63_β2m_, L65_β2m_, W29_α-chain_ and P228_α-chain_. The most visually striking feature of the binding pocket is a tunnel-like cavity that extends through the middle of the protein (S5 Fig and S2 Text). The bicyclic ring of UCB-FcRn-84 occupies this cavity, being involved in one direct and one water-mediated hydrogen bond to the main-chain of Q34_α-chain_.

As the fragment was centrally located in this region, it afforded us a tractable chemical platform to explore the binding site further (S4 Fig and S2 Text). We attempted to improve the affinity of UCB-FcRn-84 by better occupying space around the 3-fluorophenyl group, which was nested in a well-defined hydrophobic pocket. Notably, we identified a small area available for growth adjacent to the unsubstituted meta position of the 3-fluorophenyl group. After scanning a range of disubstituted phenyl derivatives, we identified 3,5 difluorophenyl as the best option, yielding a ligand with 2.4 μM affinity for the racemate. Moreover, adding a 3-pyridile group in position 5 led to an equipotent compound (UCB-FcRn-303) but with improved solubility (S5 and S6 Figs, S1 Data and S2 Text).

**Fig 2:**
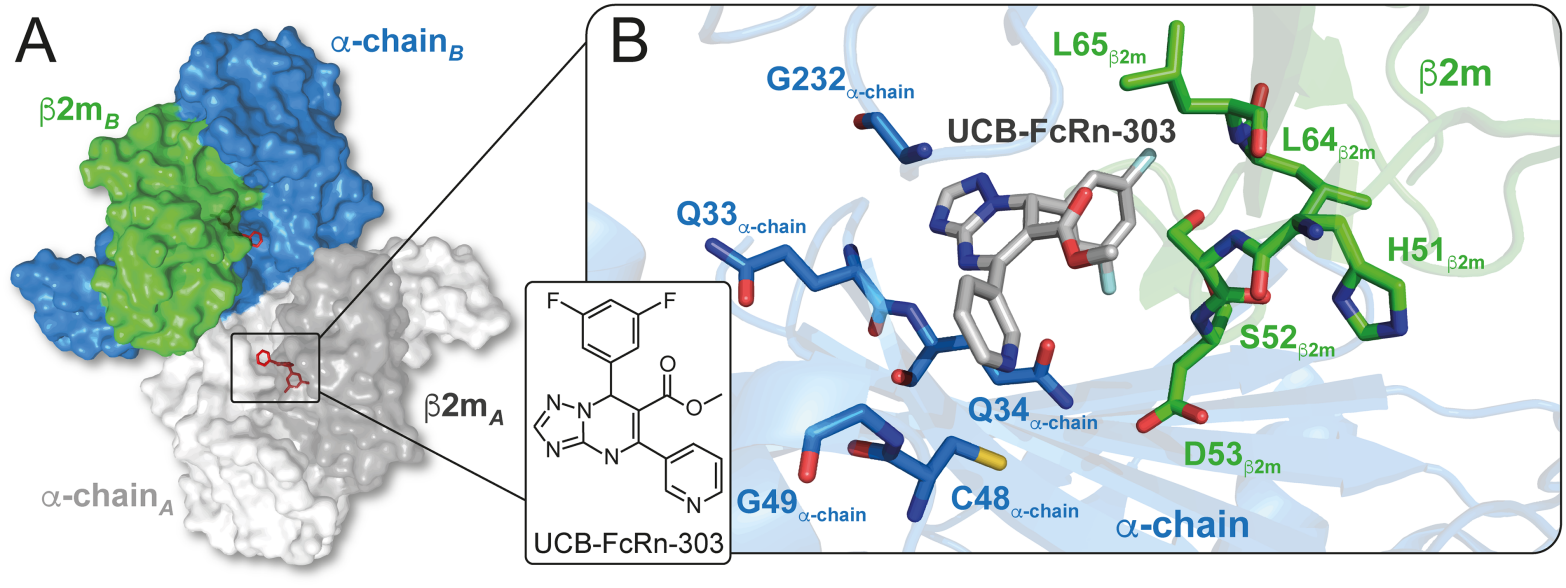
Crystal structure of the compound UCB-FcRn-303 (R enantiomer) bound to FcRn_ECD_. **(A)** The protein crystallized as a dimer composed of two β2m (dark grey and green) and two α-chain (light grey and blue) molecules. **(B)** At the interface of β2m and the α-chain, UCB-FcRn-303 (grey) occupies a binding pocket with Glycine, Cysteine, hydrophobic (Leucine), charged (Histidine, Aspartate), and polar uncharged (Serine, Glutamine) residues. β2m, β2-microglobulin; FcRn, neonatal Fc receptor; FcRn_ECD_, extracellular domain of the neonatal Fc receptor.

The crystal structure of UCB-FcRn-303 bound to FcRn_ECD_ displays the R enantiomer (Fig 2 and S5 Fig). The binding mode is consistent with the position observed for UCB-FcRn-84 (Fig 2A and 2B, S4 and S5 Figs). The di-substituted phenyl ring better fills the hydrophobic pocket in the α-chain/β2m interface, while hydrogen bond interactions are maintained with the α-chain and the local water network.

In biochemical experiments molecules from our series did not inhibit IgG binding when tested in a FcRn_ECD_–IgG FRET competition assay. This was consistent with our crystallographic studies which showed no discernible change in the IgG binding site, although accurate interpretation of small changes may be hampered by crystal contacts. The FcRn_ECD_ crystallized with two copies of the heterodimer present in the asymmetric unit, packing in an anti-parallel fashion (Fig 2A). Overlay of the IgG heavy chain–FcRn_ECD_ complex (Protein Data Bank [PDB] code 1FRT) with the ligand-free crystal structure shows significant overlap between CH2 and CH3 domains of IgG and symmetry related copies of FcRn_ECD_ (S7 Fig) [46].

In order to determine whether the ligand might induce small conformational and/or dynamic changes in regions beyond the direct binding site, we have used orthogonal solution-based techniques, which will avoid conformational restrictions imposed by crystal packing.

### Sedimented, fully protonated FcRn_ECD_ yielded well resolved proton-detected MAS NMR spectra

Until recently, solution-state and proton-detected MAS NMR typically required deuteration of proteins in the size-range of the 42 kDa FcRn_ECD_, as the signal-to-noise ratios obtained in triple-resonance experiments involving ^13^Cβ and ^13^Cα chemical-shifts critically depend on the T_2_ of ^13^C coherences [47,48]. Since we were unable to produce deuterated FcRn_ECD_ in sufficient amounts it was not possible to acquire 3D solution-state NMR spectra that would allow sequential assignments. The spectral quality of a 2D ^15^N-^1^H correlation spectrum recorded in solution, however, is remarkably high (S8 Fig) [49]. It indicates that FcRn_ECD_ most likely does not form a stable dimer of heterodimers in solution at the applied concentrations, which agrees with biochemical data and earlier studies [50]. At least 227 of the expected 349 backbone amide signals could be observed, with the remainder of the signals most probably obscured by overlap.

To circumvent these experimental limitations, we applied MAS NMR. Progress in recent years, in particular ever faster sample spinning, enables the acquisition of proton-detected MAS NMR spectra on fully protonated samples which increases sensitivity and facilitates resonance assignments through triple-resonance experiments [51–54]. At the highest MAS frequency routinely available (110 kHz), well resolved proton spectra have been reported for fully protonated microcrystalline and membrane proteins, sedimented assemblies and fibrillar proteins [55–57].

**Fig 3:**
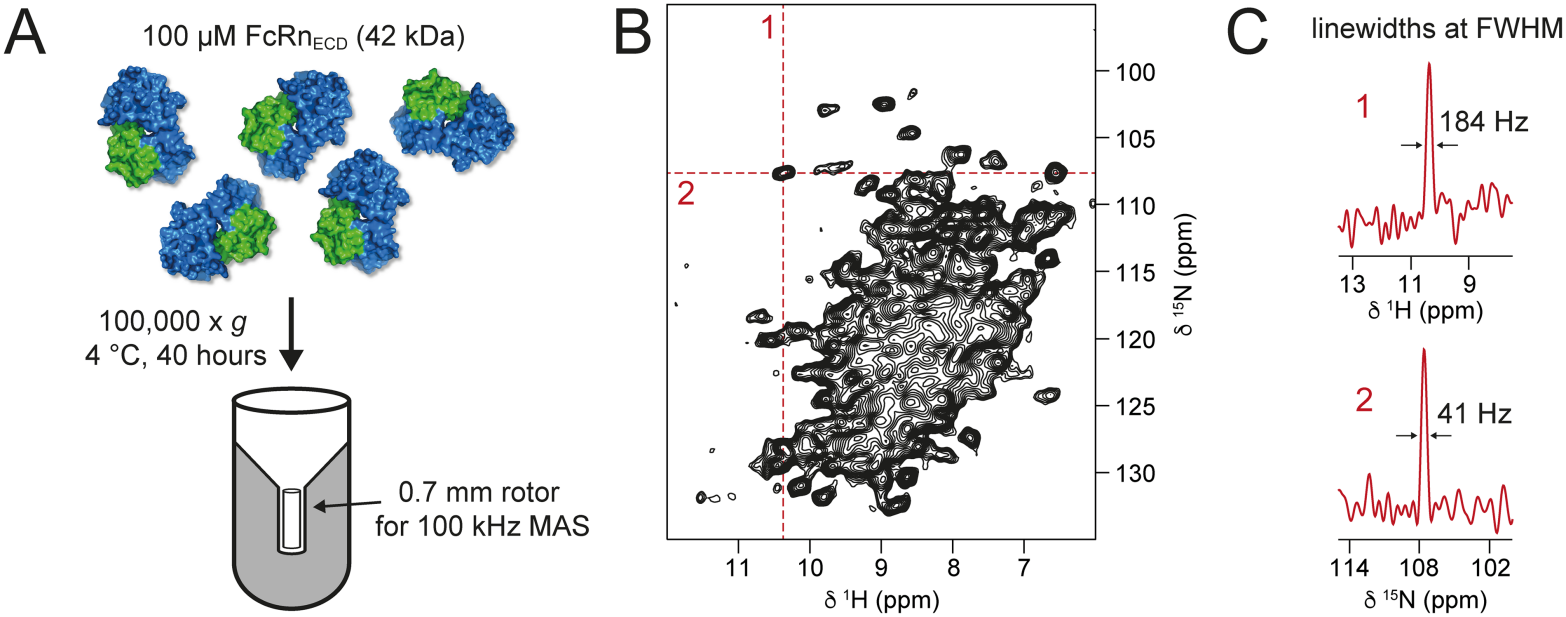
Proton-detected MAS NMR on fully protonated FcRn_ECD_. **(A)** The soluble FcRn_ECD_ (42 kDa) was sedimented by ultracentrifugation at 100,000 x *g* directly into a 0.7 mm MAS NMR rotor using a home-made filling tool. **(B)** 2D ^15^N-^1^H correlation spectrum recorded at 100 kHz MAS of fully protonated [^13^C,^15^N]-labeled FcRn_ECD_. **(C)** Typical linewidths of ^1^H (1) and ^15^N (2) at full-width-half-maximum (FWHM) of a selected cross peak from the ^15^N-^1^H spectrum. β2m, β2-microglobulin; FcRn, neonatal Fc receptor; FcRn_ECD_, extracellular domain of the neonatal Fc receptor.

In the present case, we have investigated soluble FcRn_ECD_ by MAS NMR experiments via sedimentation. For this purpose, the availability of dedicated filling tools is a prerequisite [58,59]. With the help of such appliances, we sedimented the 42 kDa soluble, fully protonated [^13^C,^15^N]-labeled FcRn_ECD_ by ultracentrifugation directly into a 0.7 mm MAS rotor (Fig 3A).

The 2D ^15^N-^1^H correlation spectrum measured at 100 kHz MAS is shown in Fig 3B. The observed linewidths demonstrate a remarkable spectral quality by MAS NMR standards (Fig 3C). Recorded at a magnetic field of about 20 T, the ^1^H and ^15^N linewidths of a selected typical cross peak are 184 Hz and 41 Hz, respectively. The amide ^1^H linewidth of FcRn_ECD_ is still higher than is observed in the model protein GB1, by a factor of approximately 1.8, but similar to the precipitated viral capsid coat protein AP205 [60]. The spectrum displays a large number of unresolved signals and a considerable fraction of signals that are well dispersed, both of which may be assigned on the basis of suitable triple-resonance spectra.

In order to monitor the folding state of FcRn_ECD_ in the sedimented sample, we compared the MAS ^15^N-^1^H spectrum to the ^15^N-^1^H correlation recorded in solution (S8 Fig and S3 Text). The agreement of the spectra indicates that the structure of FcRn_ECD_ in solution is very similar to the one in the sedimented sample. FcRn_ECD_ did not form the stable dimers of heterodimers in solution as had been observed in the crystal structure. Analytical ultracentrifugation, however, reveals a concentration-dependent protomer–diprotomer equilibrium of the FcRn_ECD_ heterodimer in solution, with a very low populated diprotomer fraction (S9 Fig and S4 Text). It is possible that this equilibrium is changed in the sedimentation process which may explain small chemical-shift differences. However, due to the signal overlap in the ^15^N-^1^H MAS NMR spectrum, a more detailed chemical-shift comparison is not possible.

### Resonance assignments in proton-detected MAS NMR spectra of FcRn_ECD_

To allow interpretation of chemical-shift perturbations (CSPs) upon binding of UCB-FcRn-303 to FcRn_ECD_, resonance assignments are critical. The high MAS frequencies now available facilitate an assignment procedure based on triple-resonance MAS NMR experiments as in solution-state NMR. These include (H)CANH, (H)CBCANH, (H)CA(CO)NH, and (H)CBCA(CO)NH spectra, yielding assignments of ^15^N, ^1^H^N^, ^13^Cα0, and ^13^Cβ chemical-shifts [61,62]. If it is not possible to obtain a (H)CBCA(CO)NH spectrum with sufficient signal-to-noise due to too short ^13^Cα and ^13^CO relaxation times, the first two experiments allow for identification of amino acids that, in a following step, can be sequentially connected according to the protein sequence by using a (H)CA(CO)NH spectrum. In this study, such spectra were acquired on the fully protonated [^13^C,^15^N]-labeled FcRn_ECD_ enabling assignments of 25 α-chain residues close to the binding pocket and of 73% of all β2m residues.

**Fig 4:**
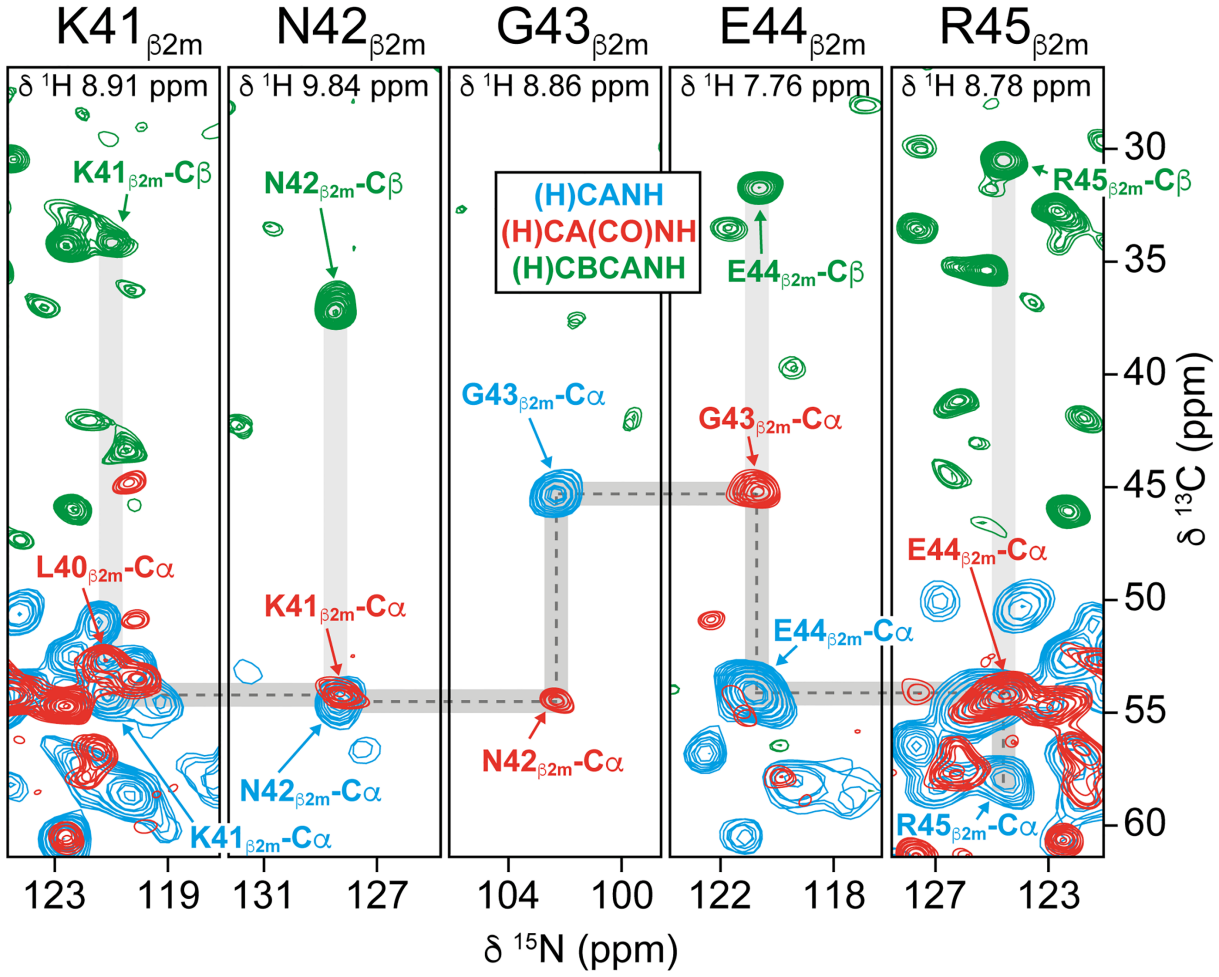
Triple-resonance MAS NMR spectra enable assignments of chemical-shifts. Sequential resonance assignments using the experiments (H)CANH (blue), (H)CA(CO)NH (red), and (H)CBCANH (green) recorded on fully protonated [^13^C,^15^N]-labeled FcRn_ECD_ at 100 kHz MAS. As an example, the sequential connections from K41_β2m_ to R45_β2m_ in β2m are indicated by dashed lines. All assigned chemical-shifts can be found in S1 Table, S2 Data, and in the BMRB (accession number 27437). β2m, β2-microglobulin; BMRB, Biological Magnetic Resonance Data Bank; FcRn_ECD_, extracellular domain of the neonatal Fc receptor; MAS, magic-angle-spinning.

To ease the assignment of β2m resonances, we made use of data in Beerbaum and colleagues, in which [^2^H,^13^C,^15^N]-labeled β2m was investigated in complex with unlabeled MHC1 [63]. However, the final assignments were achieved by establishing sequential connections along the protein backbone, as shown for the sequence from K41_β2m_ to R45_β2m_ (Fig 4). All chemical-shift assignments are listed in S1 Table and S2 Data and are deposited in the Biological Magnetic Resonance Data Bank (BMRB) (accession number 27437). In total, signals of 98 residues were assigned this way (S1 Table, S2 Data, and S5 Text). The more sensitive (H)CANH spectrum contained 84 additional resolved cross peaks that could not be assigned further due to overlap or lacking correlations in the less sensitive (H)CA(CO)NH and (H)CBCANH spectra.

Interestingly, a large number of the measured ^13^Cα, ^13^Cβ, ^15^N, and ^1^H chemical-shifts of β2m in the FcRn_ECD_ heterodimer match the observed solution-state NMR resonances for deuterated β2m in MHC1 complexes of the previous study (S1 Table and S5 Text) [63]. Differences in chemical-shifts can be explained by ^1^H/^2^H isotope effects, the limited number of amino acid substitutions in MHC1, potential dimers of FcRn_ECD_ heterodimers in the sedimented sample, and differences in buffer and temperature [64]. The similarity of the chemical-shifts supports the notion that β2m adopts its globular fold in the sedimented sample of FcRn_ECD_. Based on these findings and on the similarity between the solution-state ^15^N-^1^H and MAS ^15^N-^1^H spectra, we assume the overall FcRn_ECD_ structure to be highly similar in solution and in sedimented samples (S8 Fig, S3 Text, S1 Table and S5 Text).

In summary, (H)CANH, (H)CA(CO)NH, and (H)CBCANH spectra represent an acceptable basis for obtaining backbone resonance assignments and allowed us to exploit CSPs as monitors for structural alterations in FcRn_ECD_ upon UCB-FcRn-303 binding.

### Structural changes in FcRn_ECD_ upon binding of UCB-FcRn-303

**Fig 5:**
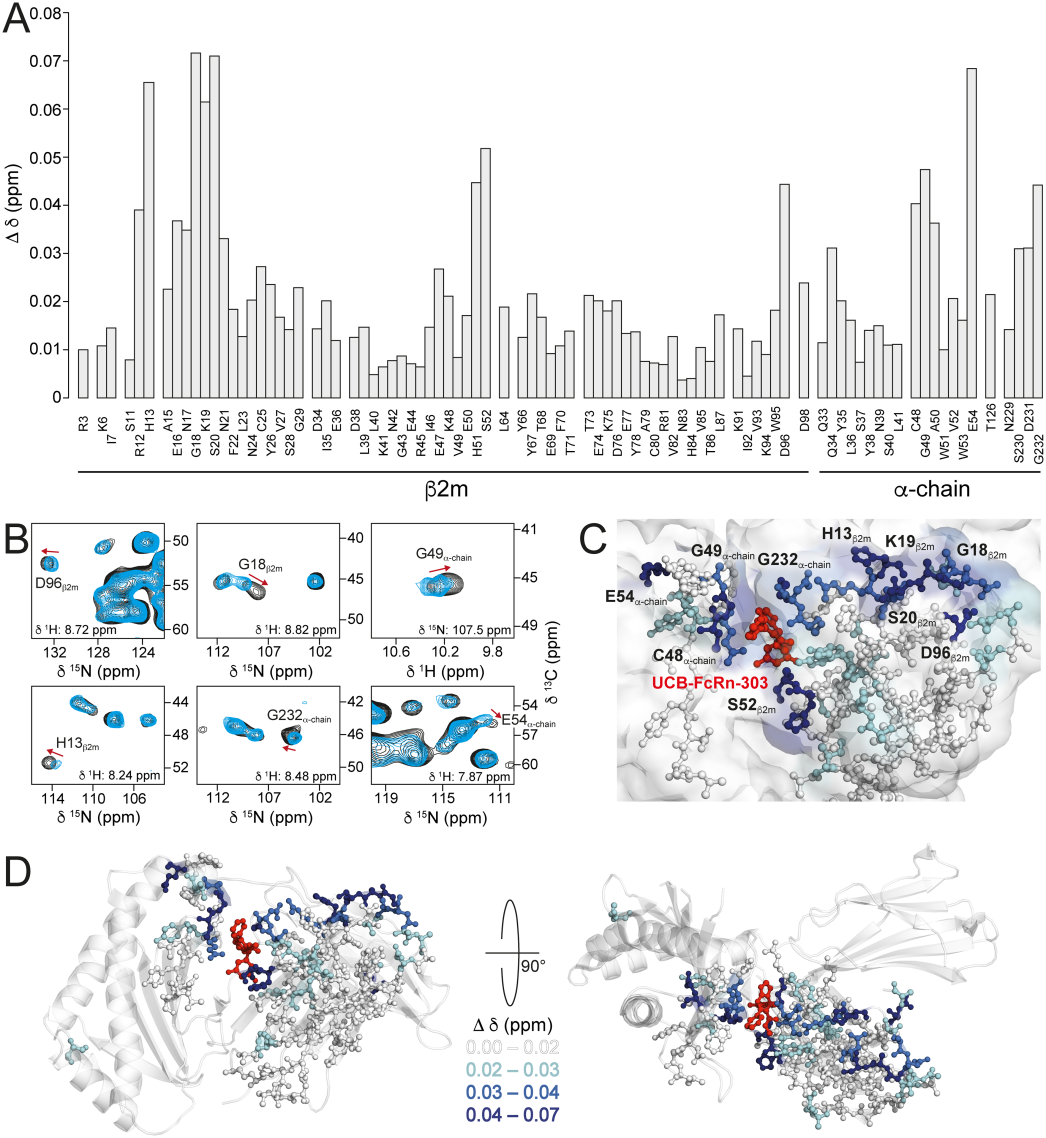
CSPs (Δδ) indicate structural changes in FcRn_ECD_ upon ligand binding. **(A)** CSPs of all assigned amino acids in FcRn_ECD_ upon binding to UCB-FcRn-303, calculated with Δδ = MIN{SQRT[(Δδ(^1^H))^2^ + (Δδ(^13^C)/10)^2^ + (Δδ(^15^N)/5)^2^]} (standard deviation = 0.015 ppm). The changes were measured in 3D (H)CANH MAS NMR spectra. **(B)** CSPs upon UCB-FcRn-303 binding to FcRn_ECD_ in 2D planes of 3D (H)CANH spectra with (black) and without (blue) the ligand, both recorded in the presence of 3% DMSO. **(C)** Structural view of UCB-FcRn-303 (red) bound to FcRn_ECD_ with assigned residues in stick representation color-coded according to their CSP (Δδ < 0.02, white; 0.02 < Δδ < 0.03, cyan; 0.03 < Δδ < 0.04, marine; 0.04 < Δδ, dark blue). **(D)** Structural view of FcRn_ECD_ in complex with UCB-FcRn-303 (*red*) with the same color-coding of changes in chemical-shifts as in (C). All chemical-shifts can be found in S1 Table, S2 Data, and in the BMRB (accession number 27437). BMRB, Biological Magnetic Resonance Data Bank; CSP, chemical-shift perturbation; FcRn, neonatal Fc receptor; FcRn_ECD_, extracellular domain of the neonatal Fc receptor.

CSPs (Δδ) are probes for both direct effects of ligand binding and concomitant long-range structural changes in receptor proteins that may hint at allosteric effects. Since differences between X-ray structures with and without ligand may be masked by crystal contacts, we analyzed this possibility by comparing 3D (H)CANH spectra recorded on samples of FcRn_ECD_ with and without UCB-FcRn-303 (Fig 5A and 5B). The introduction of a third dimension compared to a 2D ^15^N-^1^H correlation leads to better spectral resolution. The observed minimal chemical-shift differences are displayed in Fig 5A. Overall, many of the assigned signals show very similar chemical-shifts in both spectra (Δδ < 0.02 ppm, white in Fig 5C and 5D), whereas signals that shift significantly (0.02 ppm < Δδ < 0.03 ppm, cyan; 0.03 ppm < Δδ < 0.04 ppm, marine; 0.04 ppm < Δδ, dark blue in Fig 5C and 5D) appear well clustered, suggesting a conformational/dynamic change extending from the ligand binding site. In the following discussions, we consider all residues with Δδ < 0.02 ppm as not perturbed. Selected cross peaks of strongly affected residues (0.04 ppm < Δδ) are shown in 2D planes of the (H)CANH spectra with and without ligand (Fig 5B). The calculated CSPs shown in Fig 5A are based on the chemical-shift changes in all three spectral dimensions. Large CSPs displayed in Fig 5A may therefore not be obvious from the 2D planes shown in Fig 5B, see D96_β2m_.

As expected, strong effects are seen in the vicinity of the binding pocket of UCB-FcRn-303 observed by X-ray crystallography (G232_α-chain_, C48_α-chain_, G49_α-chain_ and S52_β2m_, Fig 5A and 5C) since the chemical environment of these residues is altered upon binding.

**Fig 6:**
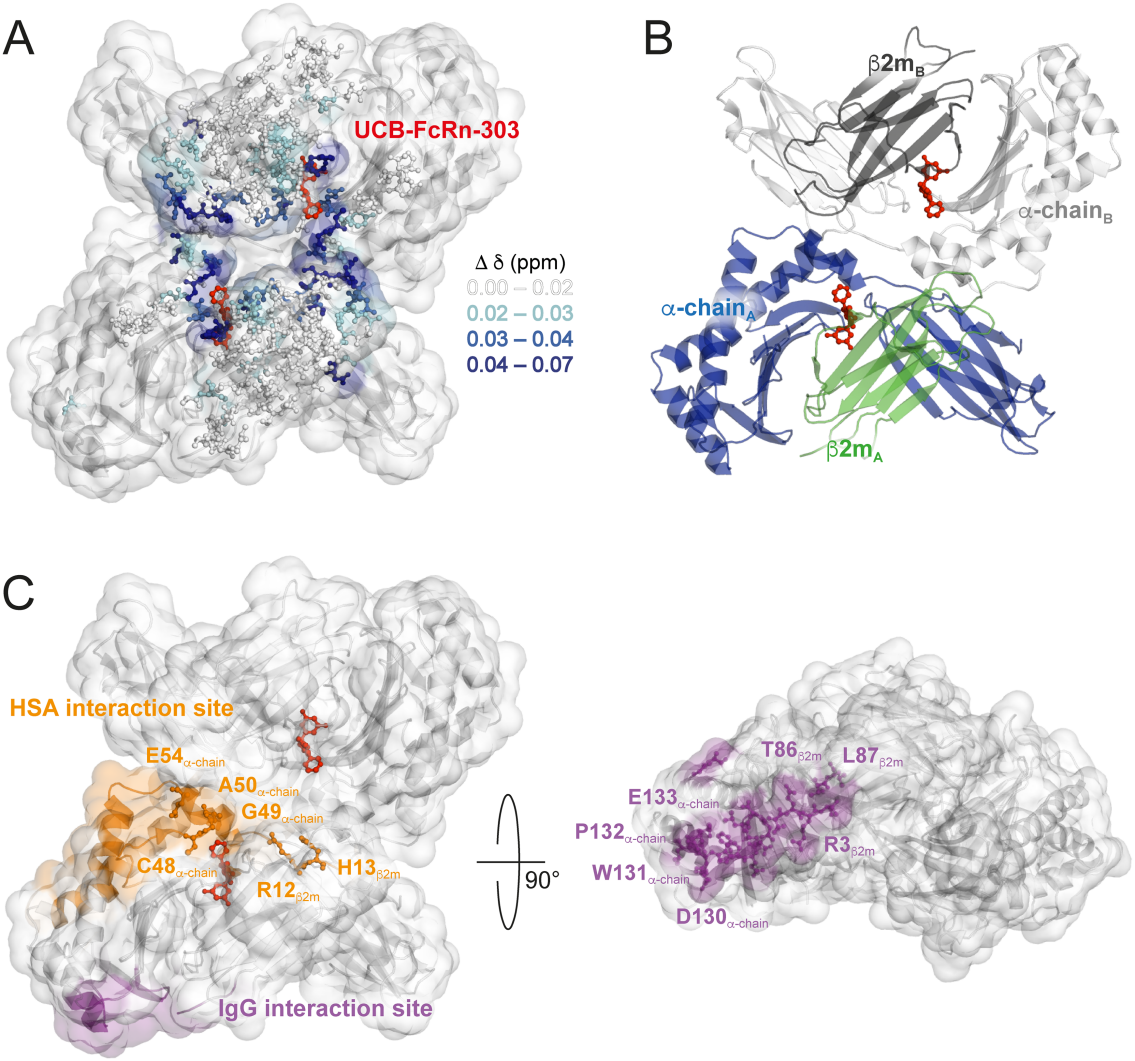
CSPs cluster at the potential FcRn_ECD_ diprotomer interface. **(A)** CSPs in surface representation of the FcRn_ECD_ diprotomer crystal structure in complex with UCB-FcRn-303 (red), with the same color-coding as in Fig 5. **(B)** For orientation, the FcRn_ECD_ crystal structure is shown in cartoon representation with β2m in green and dark grey and the α-chain molecules in blue and light grey. **(C)** The IgG and HSA interaction sites are depicted in purple and orange, respectively. The highlighted residues are discussed in the text. CSP, chemical-shift perturbation; FcRn, neonatal Fc receptor; FcRn_ECD_, extracellular domain of the neonatal Fc receptor; HSA, Human Serum Albumin; IgG, Immunoglobulin G.

More interestingly, several residues that experienced changes in their chemical-shifts are distant from the binding pocket. These include, for example, E54_α-chain_ at the C-terminal end of the short α-helix close to the binding region (Fig 5C and 5D). Such an effect could potentially be explained by a small movement of the α-helix. Furthermore, strong CSPs can be observed in a rather remote region composed of loops in β2m, involving D96_β2m_, G18_β2m_, K19_β2m_, S20_β2m_, and H13_β2m_ (Fig 5C and 5D). These residues cluster at the surface close to or at the interface of β2m and the α-chain of two different heterodimers seen in the asymmetric unit (Fig 6A and 6B). A second ligand binding site in this region of FcRn_ECD_ can be excluded since stoichiometric ratios >1 were not observed in SPR experiments or by X-ray crystallography (Fig 2A, S3, S4, S5, S6 Figs, and S1 Data). Interestingly, strong shift changes were observed at comparably long distances from the small molecule binding site. Those strong CSPs may be direct effects of ligand binding, such as introducing a slight displacement of the β2m subunit with respect to the α-chain. Alternatively, it is possible that changes in the protomer–diprotomer equilibrium occur, and thus the chemical-shifts of residues at a potential diprotomer interface are affected. Although we do not observe a stable dimer of heterodimers in solution, a small fraction is present as seen in analytical ultracentrifugation (AUC) experiments (S9 Fig and S4 Text). A diprotomer may also be more highly populated at very high protein concentrations after the applied sedimentation through ultracentrifugation in preparation of 100 kHz MAS experiments. However, the samples with and without ligand have been prepared under identical conditions, making structural effects unrelated to ligand binding unlikely. For a ligand-induced alteration of the equilibrium, long-range structural changes towards the potential diprotomer interface are required, highlighting again the possibility for the occurrence of allosteric effects upon ligand binding.

In the HSA interaction site, a number of strongly affected residues could be found. These included R12_β2m_, H13_β2m_, C48_α-chain_, G49_α-chain_, A50_α-chain_, and E54_α-chain_ with CSPs around 0.04 ppm or larger (Figs 5A, 6C and S2 Data). Some of these amino acids are close to the binding site of UCB-FcRn-303, but especially R12_β2m_, H13_β2m_, and E54_α-chain_ are distant to it and may be therefore structurally altered through allosteric effects (Figs 5C and 6C). It is possible that the FcRn–HSA interaction can be modulated with UCB-FcRn-303. The binding site to HSA, however, is located at the possible diprotomer interface and CSPs in this region may be caused by potential ligand-induced changes in the protomer–diprotomer equilibrium of FcRn_ECD_ as discussed above.

Of the NMR assigned residues, only a few from β2m are found in the IgG binding area, such as R3_β2m_ and T86_β2m_ (Fig 6C), for which negligible chemical-shift changes occur (Fig 5A) [46]. In general, residues in the β-sheet region of β2m distant from the binding site or the interface between heterodimers do not exhibit notable CSPs (Fig 5D).

In order to further investigate the potential for allosteric interference with IgG binding, we generated chemical-shift–informed overlays of the two FcRn_ECD_ crystal structures, with and without UCB-FcRn-303. All residues with Δδ < 0.02 ppm (Fig 5A) were used for fitting, including the area around R3_β2m_, T86_β2m_, and L87_β2m_ in β2m that is involved in IgG interaction (Fig 6C), yielding a root mean square deviation (r.m.s.d.) of 0.095 Å. If a movement of the α-helical region of the α-chain could be induced upon ligand binding, it may disseminate towards the loop from D130_α-chain_ to E133_α-chain_, which is part of the binding site to IgG (Fig 6C) [46]. Unfortunately, the overlay does not reveal any significant structural changes of α-chain residues close to this loop, except for subtle differences, which we are unable to isolate as a compound-induced effect due to its proximity to crystal packing interactions. Still, the CSPs observed distant to the ligand cavity reveal the potential for allosteric effects in FcRn_ECD_ induced by UCB-FcRn-303 by affecting the HSA interaction site. This provides evidence that binding of an optimized ligand could be aimed at displacing the β2m and α-chain subunits and distally disrupting the IgG binding interface of FcRn_ECD_.

### Conclusions

In the context of a druggability study, we identified a compound of <500 Da molecular weight, UCB-FcRn-303, that binds to the extracellular domain of FcRn with low μM affinity. The conserved binding site is located at the interface of β2m and the α-chain, featuring a tunnel-like cavity with solvent access from two different sides. The pocket was identified by computational chemistry methods in a theoretical druggability examination and was corroborated by fragment screening, X-ray crystallography, and NMR spectroscopy. The respective crystal structures show a dimer of heterodimers, with a 1:1 stoichiometry for the ligand/protein complex.

Due to the distance of about 35 Å between the small molecule binding pocket and the site of IgG interaction (Fig 6C), substantial allosteric effects would be required for pharmacologically relevant interference with IgG binding [46]. Such allosteric effects can be monitored by comparing X-ray structures with and without ligand and by NMR spectroscopy via the analysis of ligand-induced CSPs. We therefore established a new approach for testing ligand binding effects on soluble proteins, employing proton-detected 100 kHz MAS NMR spectroscopy on fully protonated FcRn_ECD_. After successful sedimentation of the protein by ultracentrifugation using dedicated filling tools, remarkably well-resolved proton-detected MAS NMR spectra could be obtained and partially assigned. CSPs upon UCB-FcRn-303 binding are observed around the small molecule binding pocket but also distant residues are affected, however, overlapping with the HSA interaction site.

Since a large fraction of residues in β2m towards the IgG binding site and some in the α-chain do not show substantial CSPs (Figs 5A, 6A and 6C), we generated alignments of the two X-ray structures with and without ligand, superposing the nonshifting residues. This approach revealed no substantial changes of residues close to the IgG interaction site upon FcRn-UCB-303 binding, which could modulate the FcRn_ECD_–IgG interaction. It may be envisaged that an optimized ligand could produce a shift of the α-helical region in the α-chain, with potential effects on IgG interaction. Such an optimization could include derivatives with additional functional groups that enter the α-chain/β2m interface and thus produce a slight displacement of the two with respect to each other. Strong CSPs could be observed in the HSA interaction site of FcRn_ECD_, highlighting the potential to achieve a functional modulation of FcRn with UCB-FcRn-303. However, it cannot be fully excluded that changes in oligomeric state of FcRn_ECD_ caused by the ligand under MAS conditions lead to the observed CSPs in this region.

The presented MAS NMR methodology provides an appealing approach for structural investigations of large, soluble proteins expressed in mammalian or other types of eukaryotic cells in which deuteration is challenging, especially in cases when glycosylation is crucial. Moreover, it extends the range of NMR applications to pharmacologically relevant targets, which are often inaccessible by solution-state NMR methods due to size. The MAS NMR approach works with high molecular weight targets and is independent of the physical state of the sample, providing spectral complexity can be handled, e.g., by applying appropriate labelling concepts. This facilitates protein-ligand interaction studies by NMR for any type of protein or biomolecular complex, making previously intractable pharmacological targets accessible, such as the ribosome, G protein-coupled receptors, and the like. Our investigation shows that MAS NMR complements X-ray crystallography in structure-based drug discovery campaigns. It is particularly useful to explore allosteric changes beyond small molecule binding sites, which may be difficult to observe by X-ray crystallography.

## Materials and methods

### FcRn_ECD_ vector generation

The coding sequences of extracellular domain (ECD) (amino acids 1–297) of human FcRn α-chain and human β2m were synthesized by Entelechon (Entelechon, Regensburg, Germany). The α-chain fragment was cloned into the expression vector pMH (UCB) and the β2m sequence was cloned into pMK (UCB) as HindIII and EcoRI fragments. Both vectors were digested with SalI and NotI and the relevant fragments excised and ligated to generate a vector containing both the α-chain and β2m genes (pM-ECDFcRn-B2M).

The vector was further digested with SalI and a neomycin cassette was ligated in to generate a double gene vector with antibiotic selection (pMFcRnECD-B2M-Neo).

### Generation of a FcRn_ECD_ stable mammalian cell line

HEK293 cells were transfected with pMFcRnECD-B2M-Neo using 293fectin (Thermofisher) according to the manufacturer’s instructions. Transfected cells were incubated in a static incubator at 37 °C and 8% CO_2_ for 24 hours. The cells were then diluted to the required concentration with medium supplemented with 0.5 mg/L G418 (Invitrogen) and subsequently divided into 1-mL pools in 24-well plates and incubated in a static incubator at 37 °C with 8% CO_2_. Every 7 days, the medium was removed from each well and replaced with fresh medium. After a further 14 days, the pools that exhibited cell growth were transferred to 6-well plates. These were expanded up to 50-mL cultures in E250 flasks in shaking incubators. To determine total expression of the FcRn_ECD_ heterodimer composed of β2m and α-chain, batch overgrows were set up and incubated for 10 days.

Samples of the 50-mL batch overgrow supernatants were analyzed for FcRn_ECD_ expression using western blotting. 15 μL of each supernatant was run on a (denatured Tris/Gly gel western blot) alongside known concentrations of purified human FcRn as a control.

The highest expressing pool was expanded (without Neomycin selection) up to 10-L scale in a Wave Bioreactor 20/50 EHT at 37 °C, 8% CO_2_ for 4 days, at which point the temperature was reduced to 32 °C and incubated for a further 6 days. The cell culture was harvested by centrifugation (1,000x *g* for 1 hour) and supernatant put through a 0.22-μm filter.

### Purification of FcRn_ECD_ expressed in HEK293 cells

Mammalian cell culture supernatant containing the heterodimer FcRn_ECD_ was concentrated with a 10,000 MWCO membrane using tangential flow filtration (Centramate, Pall) or Amicon stirred cell (Millipore), depending on scale. The sample buffer was exchanged into 50 mM sodium phosphate, pH 5.8, 30 mM NaCl by diluting and concentrating. FcRn_ECD_ was loaded onto a KappaSelect (GE Healthcare) column, which had been preloaded with an IgG4 monoclonal antibody to bind FcRn_ECD_, before washing with 50 mM sodium phosphate, pH 5.8, 30 mM NaCl, and eluting with 50 mM sodium phosphate, pH 8.0, 30 mM NaCl. Elution fractions were analyzed by SDS-PAGE and relevant fractions pooled.

The sample was concentrated using an Amicon spin concentrator (Millipore), 10,000 MWCO membrane. The protein was purified by Gel Filtration with a Superdex 200 (GE Healthcare) column using 25 mM sodium phosphate, pH 7.4, 100 mM NaCl. Peak fractions were analyzed by SDS-PAGE before pooling and concentrating if required.

### SPR analysis

SPR was carried out using BIAcore 4000 instruments (GE Healthcare). Reagents including CM5 sensor chips, *N-*hydroxysuccinimide (NHS), *N*-ethyl-*N*-(3-dimethylaminopropyl) carbodiimide (EDC), and ethanolamine HCl, 10 mM sodium acetate buffers (pH 5.0, pH 4.5) and HBS-P (10x buffer) were obtained from GE Healthcare.

FcRn_ECD_ was diluted into 10 mM sodium acetate buffer, pH 5.0 and immobilized on a CM5 Sensor Chip via amine coupling chemistry to a capture level of approxmimately 4,000 response units. Compounds were screened in a 10-point titration from 200 μM, at 2% DMSO, pH 6.0, and the surface was re-immobilized after each 384-well plate. Injections were performed at a flow rate of 10 μL/min.

All data were double-referenced for blank injections and reference surface, following standard procedures. Both data processing and fitting were performed using Activity Base template protocols developed in house.

### Fragment library screening using ^19^F NMR

A library of approximately 1,100 fluorine-containing fragments were cocktailed into groups of 12 ensuring no overlap of ^19^F signals. Cocktails were initially prepared at a concentration of 4.2 mM in d6-DMSO and diluted to 800 μM in PBS pH 7.4 before a final dilution to 40 μM ligand concentration (1% d6-DMSO) in either PBS containing 10% D_2_O (for control samples) or 20 μM FcRn_ECD_ containing 10% D_2_O (for protein samples). NMR spectra were acquired at 25 °C on a Bruker 600 MHz AVIII-HD spectrometer equipped with a QCI-F cryoprobe and a SampleJet autosampler. Data were collected using a CPMG pulse sequence with a total echo time of 160 ms across a sweep width of 126 ppm with an acquisition time of 1 s. All spectra were processed using TopSpin 3.2. Fragments were considered binders to FcRn_ECD_ when the ^19^F signal intensity was significantly reduced in the spectra with FcRn_ECD_ present compared to the spectra recorded in the absence of protein.

### Fragment library screening using STD NMR

STD NMR samples were prepared with a ligand to protein ratio of 50:1 (500 μM ligand, 10 μM FcRn_ECD_) in 500 μL phosphate buffered saline, pH 7.4 (90% H_2_O, 10% D_2_O) with 5% d6-DMSO to help solubilize the ligand. STD NMR spectra were recorded using a Bruker Avance III HD 600 MHz spectrometer equipped with a 5 mm QCI-F Cryoprobe. Data were acquired and processed using the standard Bruker software and were collected at 298 K. The protein was saturated in the methyl region of the spectrum at 0 ppm, and off-resonance saturation was performed at 33 ppm. A series of 120 EBurp2 pulses (50 ms each) were applied with a 4-μs delay between each pulse, resulting in total saturation time of 6 s. Protein signals were removed by applying a spinlock of 100 ms. Interleaved on- and off-resonance data were recorded, processed separately, and then the difference spectra obtained by subtracting the on- from the off-resonance spectra. Data were zero filled once and an exponential multiplication window function applied (LB 2 Hz).

### Plasmid construction of FcRn_ECD_ for expression in Sf9 insect cells

The parent construct for α-chain/β2m expresses two Open Reading Frames (ORFs) within one baculovirus multiple-target expression plasmid, pBacugs4X-1. Protein targets are based on the following amino acid sequences (both α-chain and β2m each contain their native leaders): α-chain 1–297 based on NCBI reference sequence NP_004098, β2m 1–119 based on NCBI reference sequence NP_004039. Codons for both ORFs were engineered by GeneComposer for highly expressed baculovirus genes, such that BamHI, HindIII, BglII, and EcoRI restriction sites were eliminated from the inserts to facilitate cloning [65]. Both genes, including flanking restriction sites, were synthesized at GeneArt. The ORF for α-chain was cloned behind the polyhedrin promoter via unique BamHI and HindIII sites; in this case, the BamHI site preceded the signal sequence while HindIII followed the sequence “TGAT” such that two stops are introduced after the C-terminal residue. The ORF for β2m was cloned behind the p10 promoter via unique BglII and EcoRI sites; the BglII site preceded the signal sequence, while EcoRI followed the sequence “TGATAA” such that two stops are introduced after the C-terminal residue. Using this cloning scheme, polyhedrin and p10 promoters are arranged in divergent/opposing orientations within the baculovirus transfer vector. ORFs were sequence verified prior to the commencement of expression studies.

### Expression of FcRn_ECD_ in Sf9 insect cells

The α-chain/β2m construct was transfected into Sf9 insect cells (Expression Systems) using BestBac 2.0, v-cath/chiA Deleted Linearized Baculovirus DNA (Expression Systems, Cat#-91-002). Virus from each transfection was amplified through 3 rounds to produce virus stock for large-scale production. The large-scale preparations were grown in ESF921 medium (Expression Systems, Cat#96–001). Large-scale preparations were infected using the titerless infected-cells preservation and scale-up (TIPS) method [66]. Approximately 10^6^ Tni cells (*Trichopulsia ni*, Expression Systems) per mL were infected using 1 mL of TIPS cells. Secreted proteins were harvested after 2–3 days by Tangential Flow Filtration (Spectrum KrosFlo, 0.2 μm filter Cat# P-NO2-E20U-05-N).

### Purification of FcRn_ECD_ expressed in Sf9 insect cells

Harvested baculovirus medium (*Trichoplusia ni*) containing secreted FcRn_ECD_ was concentrated 10-fold and buffer exchanged (50 mM sodium phosphate pH 5.8 and 30 mM NaCl via Tangential Flow Filtration (TFF) (Spectrum Labs). The concentrated medium was centrifuged using a JA-10 rotor at 9,000 RPM for 15 minutes at 4 °C and then filtered through a 0.2 μm bottle top filter. Three complete EDTA free protease inhibitor tablets were added to the concentrated media prior to chromatography. The filtered concentrated medium was applied to 35 mL of IgG Sepharose FF resin (GE Healthcare) equilibrated with 50 mM sodium phosphate buffer, pH 5.8, and 30 mM NaCl (Sigma) and rotated end over end for one hour at 4 °C. The resin was then poured into a gravity flow column and washed with 10 column volumes of the same equilibration buffer. FcRn_ECD_ was eluted from the resin with eight column volumes of 50 mM sodium phosphate buffer pH 8.0 and 30 mM NaCl. The elution fractions containing FcRn_ECD_ were pooled and loaded onto two 5-mL HiTrap Q FF columns (GE Healthcare) and eluted over a 1 M NaCl gradient. The fractions of interest were pooled and glycerol was added to a final concentration of 10%. The pool was concentrated to 15 mg/mL via centrifugal concentration (Amicon Regenerated Cellulose, 10 kDa MWCO, Millipore) and further purified via size exclusion chromatography over a HiLoad 16/600 Superdex 200 pg (GE Healthcare) column in 50 mM HEPES (4-(2-hydroxyethyl)-1-piperazineethanesulfonic acid) pH 7.0 and 75 mM NaCl. The fractions containing FcRn_ECD_ were pooled and concentrated for crystallography via centrifugal concentration (Amicon Regenerated Cellulose, 10 kDa MWCO, Millipore) to 9.9 mg/mL prior to being aliquoted and flash frozen in liquid nitrogen for later use in crystallization experiments.

### Crystallization of FcRn_ECD_

In order to search for crystallization conditions for FcRn_ECD_ expressed in insect cells, sitting-drop vapor diffusion crystallization trials were set up at 291 K using a variety of commercial spare-matrix (Rigaku Reagents: JCSG+, Wizard 1/2, Wizard 3/4; Hampton Research: Crystal Screen HTIndex; Molecular Dimensions: PACT, Morpheus, Proplex; Microlytics: MCSG1) using 0.4 μL of protein solution at 5 mg/mL that were mixed with 0.4 μL of reservoir solution and equilibrated against 80 μL of reservoir solution. The initial crystallization trials produced small kite crystals in several conditions that contain PEG 3350, PEG 6000, or PEG 8000 at low pH. Low pH crystals of FcRn_ECD_ were produced in an optimized crystallization condition screen (Rigaku) containing 12%–16% PEG 6000, 100 mM Citric Acid/Ammonium citrate tribasic pH 3.00–3.09. Crystals of FcRn_ECD_ appeared within 24 hours and grew larger overtime, typically to 50–150 microns in size. The crystals were harvested using 20% glycerol as a cryoprotectant and flash frozen in liquid nitrogen prior to data collection.

Additionally, small rod-like crystals appeared in a single condition at a significantly higher pH (condition PACT H3, 100 mM Bis-Tris Propane/HCl pH 8.5, 200 mM NaI, and 20% PEG 3350) [67]. High/neutral pH crystals of FcRn_ECD_ were produced in optimized crystallization condition containing 100 mM Bis-Tris Propane/HCl pH 8.5, 200 mM NaI, and 20% PEG 3350. Crystals of FcRn_ECD_ appeared within 48 hours and grew larger overtime, typically greater than 150 microns in size. The crystals were harvested using 20% ethylene glycol and flash frozen in liquid nitrogen prior to data collection. To obtain compound bound crystals, apo FcRn_ECD_ crystals grown at pH 3 were soaked for three days in buffer containing 0.1 M Citric Acid/NaOH at pH 3.0, 20% w/v PEG 6000, 20% glycerol, and 12.5–20 mM compound dissolved in 100% DMSO. For crystallization of the UCB-FcRn-303 bound structure of FcRn_ECD_, protein expressed in Sf9 insect cells was used.

### Structure determination by X-ray crystallography

Datasets were collected at Canadian Light Source (CLS) on beamline 08ID-1 (CMCF) equipped with a Rayonix MX300 CCD X-ray detector and Advanced Photon Source (APS) on beamline 21-ID-F (LS-CAT) equipped with a Marmosaic 225 CCD X-ray detector. Diffraction data were reduced and scaled with XDS/XSCALE [68]. The structures of FcRn_ECD_ at low and high/neutral pH were solved by molecular replacement using a pre-existing structure of the complex. A significant portion of the structure required remodeling; therefore, Phenix.autobuild was run to generate a starting structure for further refinement. All structures were refined using iterative cycles of TLS and restrained refinement with Phenix.refine and model-building using the Crystallographic Object-Oriented Toolkit (COOT; Version 0.8.1-pre) and were validated using Molprobity prior to deposition in the PDB (IDs 6C97, 6C98, 6C99) [69–73]. Diffraction data and refinement statistics for apo FcRn_ECD_ at pH 3 and both ligand bound structures are listed in S2 Table.

### Molecular dynamics simulation

The FcRn_ECD_ structures at pH 3 and pH 8.5 were used as the starting points for molecular dynamics simulation, after adding hydrogen. Protonation states and missing loops were predicted with Maestro (Schrödinger LLC). The structure was solvated in a dodecahedron such that no protein atom was within 10 Å of the edge of the solvent. Monatomic ions were added to a salt concentration of 0.15 M. All simulations were 1,000 ns in length and were carried out with GROMACS 4.6.2 [74]. Particle mesh Ewald was used for long-range electrostatics along with 10 Å cutoffs for Coulomb and Lennard–Jones potential functions.

### Expression of fully protonated [^13^C,^15^N]-labeled FcRn_ECD_ for NMR experiments

FcRn_ECD_ was expressed using a stable HEK293 cell line, as described above, but the growth media was replaced with Bioexpress 6000 media (Cambridge Isotope Laboratories).

### Acquisition of 2D ^15^N-^1^H TROSY solution-state NMR spectrum on fully protonated [^13^C,^15^N]-labeled FcRn_ECD_

The solution-state 2D ^15^N-^1^H TROSY spectrum was acquired from a 0.35-mL sample of 300 μM [^13^C,^15^N]-labeled FcRn_ECD_ in a 25 mM Na_2_HPO_4_, 100 mM NaCl, 50 μM EDTA, 0.02% (w/v) NaN_3_ buffer at pH 7.4 containing 5% D_2_O/95% H_2_O [49]. NMR data were acquired at 35 °C on a 600 MHz Bruker AVIII HD spectrometer fitted with a cryogenically cooled probe. The spectrum was acquired for 40 minutes with acquisition times of 40 ms in F_1_ (^15^N) and 60 ms in F_2_ (^1^H). It was processed using Topspin 3.5 (Bruker Biospin Ltd) with linear prediction used to extend the effective acquisition time to 60 ms in F_1_.

### Sedimentation of FcRn_ECD_ by ultracentrifugation for NMR experiments using fast MAS

A 0.5-mL sample of 100 μM [^13^C,^15^N]-labeled FcRn_ECD_ in a 25 mM Na_2_HPO_4_, 100 mM NaCl, 50 μM EDTA, 0.02% (w/v) NaN_3_ buffer at pH 7.4 containing 3% DMSO was directly ultracentrifuged into a 0.7 mm MAS NMR rotor at 100,000 x *g* and 4 °C for 40 hours using a swinging bucket ultracentrifuge rotor. Dedicated home-made filling tools were used for this step. Both bottom and top caps of the 0.7 mm MAS rotor were glued to avoid the loss of liquid or removal of caps during MAS. Excess protein and liquid after ultracentrifugation was removed before closing the 0.7 mm NMR MAS rotor. For experiments to detect CSPs, 3 mM UCB-FcRn-303 was added to a 0.5-mL sample of 100 μM [^13^C,^15^N]-labeled FcRn_ECD_ in a 25 mM Na_2_HPO_4_, 100 mM NaCl, 50 μM EDTA, 0.02% (w/v) NaN_3_ buffer at pH 7.4 containing 3% DMSO (ligand:protein ratio of 30:1). The ligand-bound sample was sedimented into a second 0.7 mm MAS rotor under the same conditions as the ligand-free sample.

### Proton-detected NMR experiments using fast MAS on fully protonated [^13^C,^15^N]-labeled FcRn_ECD_

All MAS NMR experiments were performed on a Bruker AVANCE III 850 MHz spectrometer. The spectra were recorded at 80, 90, or 100 kHz MAS with a triple-resonance 0.7 mm MAS probe (Bruker) and referenced to 4,4-dimethyl-4-silapentane-1-sulfonic acid (DSS) (see S3 Table for experimental parameters). The sample temperature of 283 K was monitored by the frequency of the water resonance line. To achieve this sample temperature, the sample was cooled during MAS using nitrogen gas through a cooling unit (BCU II, Bruker) in strong mode with the gas flow set to 400 L/h. (H)NH, (H)CANH, (H)CA(CO)NH, and (H)CBCANH spectra were recorded using Cross Polarization (CP) (heteronuclear transfers) and DREAM (homonuclear transfers) magnetization transfer steps according to the procedures described in Penzel and colleagues [61,75,76]. All spectra were processed with Topspin 3.5 (Bruker) and analyzed with CCPNmr Analysis v. 2.4.2. [77]. All assigned chemical-shifts and observed CSPs are listed in S1 Table and S2 Data, and are deposited in the Biological Magnetic Resonance Data Bank (BMRB) (accession number 27437).

### Analytical ultracentrifugation of FcRn_ECD_

Sedimentation velocity experiments were performed at 8 °C and 35,000 rpm with an An-60Ti rotor using 12-mm Epon 2-sector centerpieces. For each of the three measurements at different FcRn_ECD_ concentrations (4 μM, 14 μM, and 52 μM), a 400-μL sample in 10 mM Na_2_HPO_4_, 137 mM NaCl, 2.7 mM KCl, 1.8 mM KH_2_PO_4_ buffer at pH 7.2 was used. The data were analyzed and plotted with the GUSSI implementation and SEDFIT [78].

### Experimental and analytical details for synthetic analogues

All solvents and reagents were used as received from commercial suppliers, unless noted otherwise. The compounds were named using the Biovia Draw 2016 package (IUPAC).

NMR spectra were recorded on a Bruker Avance III HD 500 MHz or 250 MHz spectrometer.

The chemical-shifts (δ) reported are given in parts per million (ppm) and the coupling constants (J) are in Hertz (Hz). The spin multiplicities are reported as s = singlet, d = doublet, t = triplet, q = quartet, dd = doublet of doublet, ddd = doublet of doublet of doublet, dt = doublet of triplet, td = triplet of doublet, and m = multiplet.

uPLC-MS was performed on a Waters Acquity UPLC system coupled to a Waters Acquity PDA detector, an ELS detector and an MSD (Scan Positive: 150–850). Method (pH 3): Phenomenex Kinetix-XB C18 (2.1 x 100 mm, 1.7 μm) column. Elution with a linear gradient of Water + 0.1% Formic acid and Acetonitrile + 0.1% Formic acid at a flow rate of 0.6 mL/min. Chiral SFC analysis: Waters Thar 3100 SFC system connected to Waters 2998 PDA detector, Chiralcel OD-H 25 cm. Chiral SFC separation: Water Thar SFC system with a Waters Thar FDM pump, Waters Thar Alias autoinjector, Waters Thar fraction collector and a Waters 2998 PDA detector.

#### *(R)* and *(S)* 1-[7-(3-Fluorophenyl)-5-methyl-4,7-dihydro-[1,2,4]triazolo[1,5-a]pyrimidin-6-yl]ethanone (UCB-FcRn-84) (S10 Fig)

1-[7-(3-fluorophenyl)-5-methyl-4,7-dihydro-[1,2,4]triazolo[1,5-a]pyrimidin-6-yl]ethanone (CAS 691368-95-3) was purchased as a racemate from Life Chemicals and the mixture separated by chiral chromatography using a Chiralpak AD phase (100*500), 300 mL/min with an heptane/isopropanol (8/2) system. 1.21 G of starting material led to respectively 577 mg and 588 mg of separated isomers. Chiral analytical SFC: RT = 8.22 min, 100% ee; RT = 10.40 min, 100% ee.

#### 1-[7-(3,5-Difluorophenyl)-5-methyl-4,7-dihydro-[1,2,4]triazolo[1,5-a]pyrimidin-6-yl]ethanone (S11 Fig)

A stirred solution of 3,5-difluorobenzaldehyde (0.1 mL, 0.946 mmol), pentane-2,4-dione (0.146 mL, 1.42 mmol, 1.5 eq.), and *4H*-1,2,4-triazol-3-amine (119 mg, 1.42 mmol, 1.5 eq.) in N,N-dimethylformamide (1.5 mL) was irradiated in a microwave oven (up to 200 W) at 150 °C for 60 min. The reaction mixture was left to cool down to ambient temperature and water (6 mL) was added leading to the formation of a precipitate. The resulting solid was collected by filtration, rinsed with water (2 x 1 mL) and cyclohexane (2 x 1 mL), then triturated in hot acetonitrile (1 mL) and dried *in vacuo* to afford 94 mg (34% yield) of the title compound as a pale yellow solid.

The ^1^H NMR analysis yielded (500 MHz, DMSO-d6) δ 10.89 (s, 1H), 7.70 (s, 1H), 7.14 (t, J = 9.1 Hz, 1H), 6.98 (d, J = 6.3 Hz, 2H), 6.46 (s, 1H), 2.46 (s, 3H), 2.20 (s, 3H). uPLC-MS: [M+H]^+^ m/z = 291, RT = 2.38 min (99%).

#### *(R)* and *(S)* 1-[7-(3,5-Difluorophenyl)-5-methyl-4,7-dihydro-[1,2,4]triazolo[1,5-a]pyrimidin-6-yl]ethanone (S12 Fig)

The racemate (50 mg) was separated by chiral preparative chromatography on a Chiralpak ASV (50*490) phase, 80 mL/min with a heptane/ethanol (9/1) system to afford 24 mg and 19 mg of the pure enantiomers. Chiral analytical SFC: RT = 10.89 min, 100% ee; RT = 15.16 min, 97.8% ee.

#### Methyl 7-(3,5-difluorophenyl)-5-(3-pyridyl)-4,7-dihydro-[1,2,4]triazolo[1,5-a]pyrimidine-6-carboxylate (UCB-FcRn-303) (S13 Fig)

To a stirred solution of 3,5-difluorobenzaldehyde (150 mg, 1.06 mmol), methyl 3-oxo-3-(pyridin-3-yl)propanoate (265 mg, 1.48 mmol, 1.4 eq.), and *4H*-1,2,4-triazol-3-amine (124 mg, 1.48 mmol, 1.4 eq.) in N,N-dimethylformamide (1.5 mL) was added chloro(trimethyl)silane (0.268 mL, 2.11 mmol) dropwise. The reaction mixture was then irradiated in a microwave oven (up to 200 W) at 130 °C for 60 minutes. The reaction mixture was left to cool down to ambient temperature and water (6 mL) was added leading to the formation of a precipitate. The resulting solid was collected by filtration, rinsed with water (2 x 1 mL) and cyclohexane (2 x 1 mL) then recrystallized from acetonitrile (2 mL) and dried in vacuo to afford 238 mg (59% yield) of the title compound as a pale yellow solid. The ^1^H NMR analysis yielded (500 MHz, DMSO-d6) δ 11.18 (s, 1H), 8.67–8.61 (m, 2H), 7.92 (dt, J = 7.8, 1.9 Hz, 1H), 7.75 (s, 1H), 7.48 (dd, J = 7.8, 4.9 Hz, 1H), 7.20 (tt, J = 9.2, 2.2 Hz, 1H), 7.16–7.10 (m, 2H), 6.50 (s, 1H), 3.27 (s, 3H). uPLC-MS: [M+H]^+^ m/z = 370, RT = 2.12 min (97%).

## Supporting information

S1 Fig Ligandability assessments on FcRn_ECD_ and predicted small molecule binding sites. At low pH binding sites with the capacity to yield high affinity binding are restricted to the dimer interface, with the region described as either one large or three distinct pockets (pockets A-C). At neutral and basic pH, transient sites arise at the albumin binding site, between the α1 and α2 helices (D), and between the β2m and the α3 domain (E). β2m, β2-microglobulin; FcRn_ECD_, extracellular domain of the neonatal Fc receptor.
(JPG)

S2 Fig Evolutionary conservation of FcRn_ECD_. **(A)** The pH 3 structure of the human FcRn_ECD_ heterodimer is colored to illustrate sequence conservation in vertebrate orthologues. Universally conserved residues are colored white; mutated residues are shown in red, with the color intensity indicating the BLOSUM62 score of the worst-matching substitution (darker red = more radical amino acid change away from the human residue). Species included in the analysis are: *Pan troglodytes*, *Gorilla gorilla*, *Pongo pygmaeus*, *Macaca mulatta*, *Callithrix aurita*, *Microcebus murinus*, *Otolemur garnettii*, *Mus musculus*, *Rattus norvegicus*, *Cavia porcellus*, *Oryctolagus cuniculus*, and *Bos taurus*. Mutations occur throughout the α-chain and β2m. Areas of clear conservation include the interface of α-chain and β2m and the central cavity that was detected in the SiteMap analysis. **(B)** For reference, human FcRn_ECD_ from the HSA-bound FcRn structure (PDB code 4N0F) has been colored to highlight residues that constitute the surfaces with the Fc moiety of IgG (magenta) and HSA (orange), the α-chain is shown in green and β2m in cyan. β2m, β2-microglobulin; FcRn, neonatal Fc receptor; FcRn_ECD_, extracellular domain of the neonatal Fc receptor; HSA, Human Serum Albumin; IgG, Immunoglobulin G; PDB, Protein Data Bank.
(TIF)

S3 Fig UCB-FcRn-84 binds with K_D_ approximately 80 μM to FcRn_ECD_ as measured by SPR. The numerical values can be found in S1 Data. FcRn, neonatal Fc receptor; FcRn_ECD_, extracellular domain of the neonatal Fc receptor; SPR, Surface Plasmon Resonance.
(TIF)

S4 Fig Crystal structure of the compound UCB-FcRn-84 bound to FcRn_ECD_. The compound binds at the interface of β2m (green) and the α-chain (blue). Also in this crystal structure, a second heterodimer can be found in the asymmetric unit (grey). β2m, β2-microglobulin; FcRn, neonatal Fc receptor; FcRn_ECD_, extracellular domain of the neonatal Fc receptor.
(JPG)

S5 Fig Crystal structure of UCB-FcRn-303 bound to FcRn_ECD_. The compound occupies the same binding pocket as UCB-FcRn-84 at the interface of β2m (green) and the α-chain (blue). The binding region is a tunnel-like cavity extending through the protein. Again, a second heterodimer is found in the crystal structure (depicted in grey). β2m, β2-microglobulin; FcRn, neonatal Fc receptor; FcRn_ECD_, extracellular domain of the neonatal Fc receptor.
(JPG)

S6 Fig UCB-FcRn-303 binds with K_D_ = 2.4 μM to FcRn_ECD_ as measured by SPR. The numerical values can be found in S1 Data. FcRn, neonatal Fc receptor; FcRn_ECD_, extracellular domain of the neonatal Fc receptor; SPR, Surface Plasmon Resonance.
(TIF)

S7 Fig The CH2 and CH3 domains (red) of IgG heavy chain in complex with FcRn_ECD_ (α-chain in blue, β2m in green) (PDB code 1FRT) show overlap with symmetry related copies (dark and medium grey) of ligand-free FcRn_ECD_ (light grey) [46]. β2m, β2-microglobulin; FcRn_ECD_, extracellular domain of the neonatal Fc receptor; IgG, Immunoglobulin G; PDB, Protein Data Bank.
(JPG)

S8 Fig Comparison of FcRn_ECD_^15^N-^1^H NMR spectra in solution and after sedimentation. **(A)** 2D ^15^N-^1^H correlation using TROSY of fully protonated [^13^C,^15^N]-labeled FcRn_ECD_ measured in solution. **(B)** Overlay of the spectrum shown in (A) (orange) with a 2D ^15^N-^1^H spectrum of sedimented fully protonated [^13^C,^15^N]-labeled FcRn_ECD_ recorded at 100 kHz MAS (black). FcRn_ECD_, extracellular domain of the neonatal Fc receptor; MAS, magic-angle-spinning.
(JPG)

S9 Fig Analytical ultracentrifugation of FcRn_ECD_. Sedimentation velocity experiments at three different concentrations (52 μM, grey; 14 μM, red; 4 μM, blue) exhibit protein concentration dependent peaks at 3.5 S, 5.1 S, and 5.3 S. FcRn_ECD_, extracellular domain of the neonatal Fc receptor.
(JPG)

S10 Fig *(R)* and *(S)* 1-[7-(3-Fluorophenyl)-5-methyl-4,7-dihydro-[1,2,4]triazolo[1,5-a]pyrimidin-6-yl]ethanone (UCB-FcRn-84). FcRn, neonatal Fc receptor.
(PDF)

S11 Fig 1-[7-(3,5-Difluorophenyl)-5-methyl-4,7-dihydro-[1,2,4]triazolo[1,5-a]pyrimidin-6-yl]ethanone. (PDF)

S12 Fig *(R)* and *(S)* 1-[7-(3,5-Difluorophenyl)-5-methyl-4,7-dihydro-[1,2,4]triazolo[1,5-a]pyrimidin-6-yl]ethanone. (PDF)

S13 Fig Methyl 7-(3,5-difluorophenyl)-5-(3-pyridyl)-4,7-dihydro-[1,2,4]triazolo[1,5-a]pyrimidine-6-carboxylate (UCB-FcRn-303). FcRn, neonatal Fc receptor.
(PDF)

S1 Data Numerical values of SPR experiments in S3 and S6 Figs. (XLSX)

S2 Data Observed chemical-shifts and CSP values of FcRn_ECD_ with and without UCB-FcRn-303 as shown in Fig 5. β2m, β2-microglobulin; CSP, chemical-shift perturbation; FcRn, neonatal Fc receptor; FcRn_ECD_, extracellular domain of the neonatal Fc receptor; MHC1, class I major histocompatibility complex.
(XLSX)

S1 Table ^1^H, ^15^N, ^13^Cα, and ^13^Cβ chemical-shifts observed in proton-detected NMR experiments at 100 kHz MAS on sedimented fully protonated [^13^C,^15^N]-labeled FcRn_ECD_. They are compared to the corresponding chemical-shifts (Beerbaum and colleagues) of [^2^H,^13^C,^15^N]-labeled β2m in MHC1 complexes measured in solution-state NMR [63]. Amino acids of the α-chain are depicted in blue, β2m residues in green. β2m, β2-microglobulin; FcRn_ECD_, extracellular domain of the neonatal Fc receptor; MAS, magic-angle-spinning.
(PDF)

S2 Table X-ray diffraction data and refinement statistics. (PDF)

S3 Table Experimental parameters for proton-detected MAS NMR experiments on fully protonated [^13^C,^15^N]-labeled FcRn_ECD_. FcRn_ECD_, extracellular domain of the neonatal Fc receptor; MAS, magic-angle-spinning.
(PDF)

S1 Text Ligandability assessments on FcRn_ECD_. FcRn_ECD_, extracellular domain of the neonatal Fc receptor.
(PDF)

S2 Text UCB-FcRn-303 binds in a tunnel-like cavity with low μM affinity. FcRn, neonatal Fc receptor.
(PDF)

S3 Text FcRn_ECD_ adopts a similar structure in solution and sedimented samples. FcRn_ECD_, extracellular domain of the neonatal Fc receptor.
(PDF)

S4 Text Analytical ultracentrifugation reveals a small fraction of dimers of heterodimers at higher concentrations of FcRn_ECD_ in solution. FcRn_ECD_, extracellular domain of the neonatal Fc receptor.
(PDF)

S5 Text Observed chemical-shifts of FcRn_ECD_ in MAS NMR experiments. FcRn, neonatal Fc receptor; MAS, magic-angle-spinning.
(PDF)

## Abbreviations

β2m: β2-microglobulin
APS: Advanced Photon Source
AUC: analytical ultracentrifugation
BMRB: Biological Magnetic Resonance Data Bank
CD8: cluster of differentiation 8
CLS: Canadian Light Source
COOT: Crystallographic Object-Oriented Toolkit
CP: Cross Polarization
CSP: chemical-shift perturbation
ECD: extracellular domain
FcRn: neonatal Fc receptor
FcRn_ECD_: extracellular domain of the neonatal Fc receptor
HSA: Human Serum Albumin
IgG: Immunoglobulin G
MAS: magic-angle-spinning
MHC1: class I major histocompatibility complex
ORF: Open Reading Frame
PDB: Protein Data Bank
r.m.s.d.: root mean square deviation
SPR: Surface Plasmon Resonance
TCR: T cell receptor
TIPS: titerless infected-cells preservation and scale-up
