# Supplementary figures and images for: Insight into small molecule binding to the neonatal Fc receptor by X-ray crystallography and 100 kHz magic-angle-spinning NMR
Source: PLoS Biol. 2018 May 21;16(5):e2006192. doi: 10.1371/journal.pbio.2006192 (PMC5983862; doi:10.1371/journal.pbio.2006192)

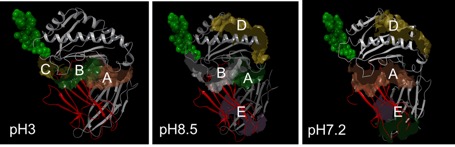

Supplement: S1 Fig — At low pH binding sites with the capacity to yield high affinity binding are restricted to the dimer interface, with the region described as either one large or three distinct pockets (pockets A-C). At neutral and basic pH, transient sites arise at the albumin binding site, between the α1 and α2 helices (D), and between the β2m and the α3 domain (E). β2m, β2-microglobulin; FcRnECD, extracellular domain of the neonatal Fc receptor. (JPG) [file pbio.2006192.s001.jpg]

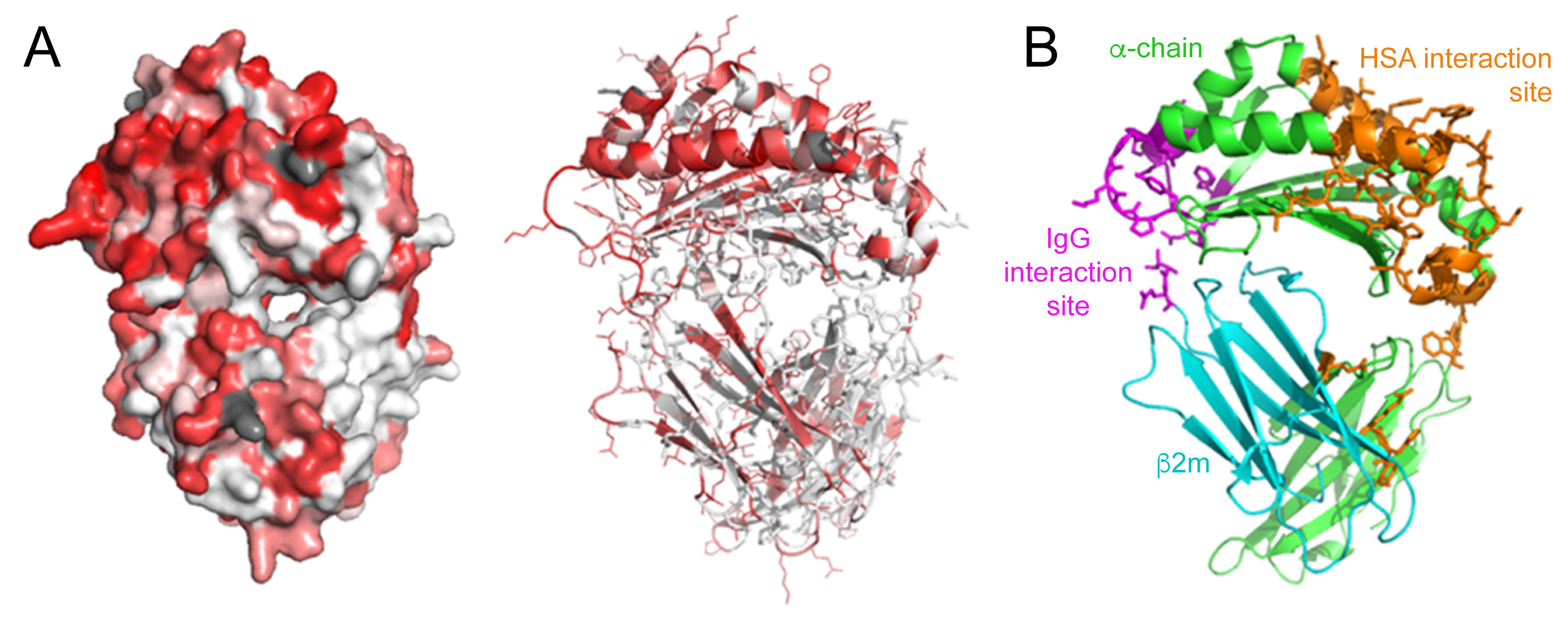

Supplement: S2 Fig — (A) The pH 3 structure of the human FcRnECD heterodimer is colored to illustrate sequence conservation in vertebrate orthologues. Universally conserved residues are colored white; mutated residues are shown in red, with the color intensity indicating the BLOSUM62 score of the worst-matching substitution (darker red = more radical amino acid change away from the human residue). Species included in the analysis are: Pan troglodytes, Gorilla gorilla, Pongo pygmaeus, Macaca mulatta, Callithrix aurita, Microcebus murinus, Otolemur garnettii, Mus musculus, Rattus norvegicus, Cavia porcellus, Oryctolagus cuniculus, and Bos taurus. Mutations occur throughout the α-chain and β2m. Areas of clear conservation include the interface of α-chain and β2m and the central cavity that was detected in the SiteMap analysis. (B) For reference, human FcRnECD from the HSA-bound FcRn structure (PDB code 4N0F) has been colored to highlight residues that constitute the surfaces with the Fc moiety of IgG (magenta) and HSA (orange), the α-chain is shown in green and β2m in cyan. β2m, β2-microglobulin; FcRn, neonatal Fc receptor; FcRnECD, extracellular domain of the neonatal Fc receptor; HSA, Human Serum Albumin; IgG, Immunoglobulin G; PDB, Protein Data Bank. (TIF) [file pbio.2006192.s002.tif]

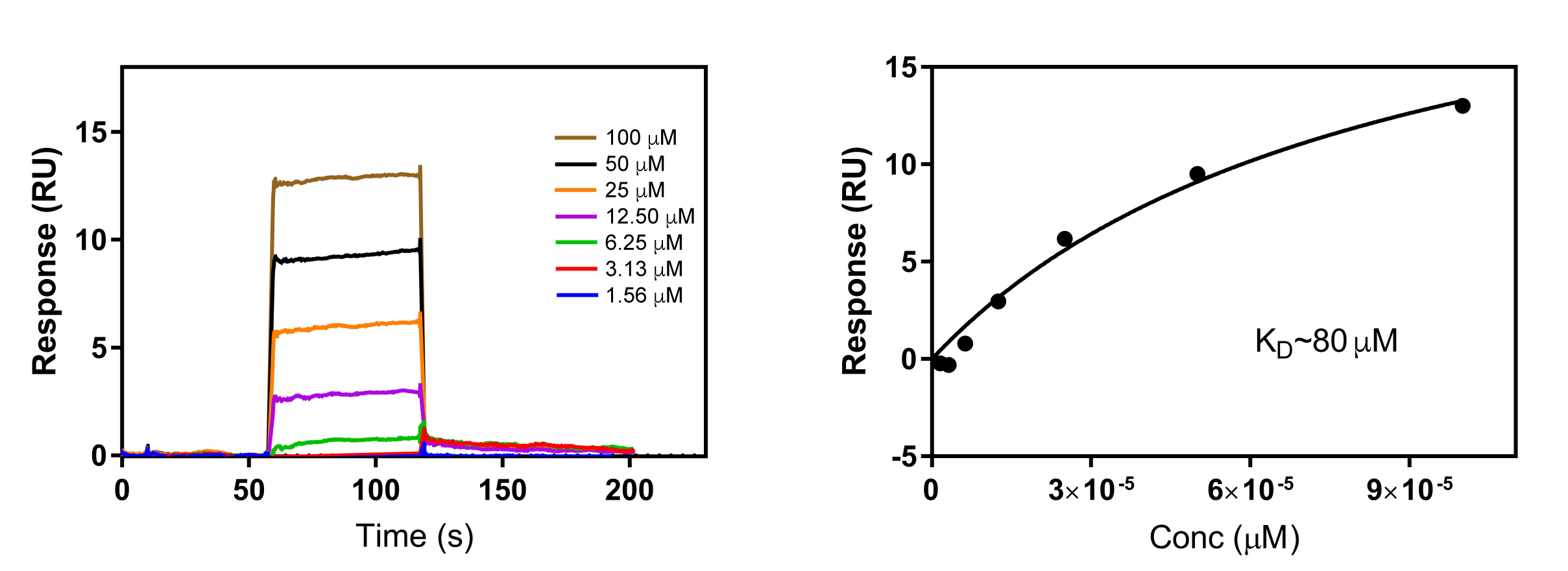

Supplement: S3 Fig — The numerical values can be found in S1 Data. FcRn, neonatal Fc receptor; FcRnECD, extracellular domain of the neonatal Fc receptor; SPR, Surface Plasmon Resonance. (TIF) [file pbio.2006192.s003.tif]

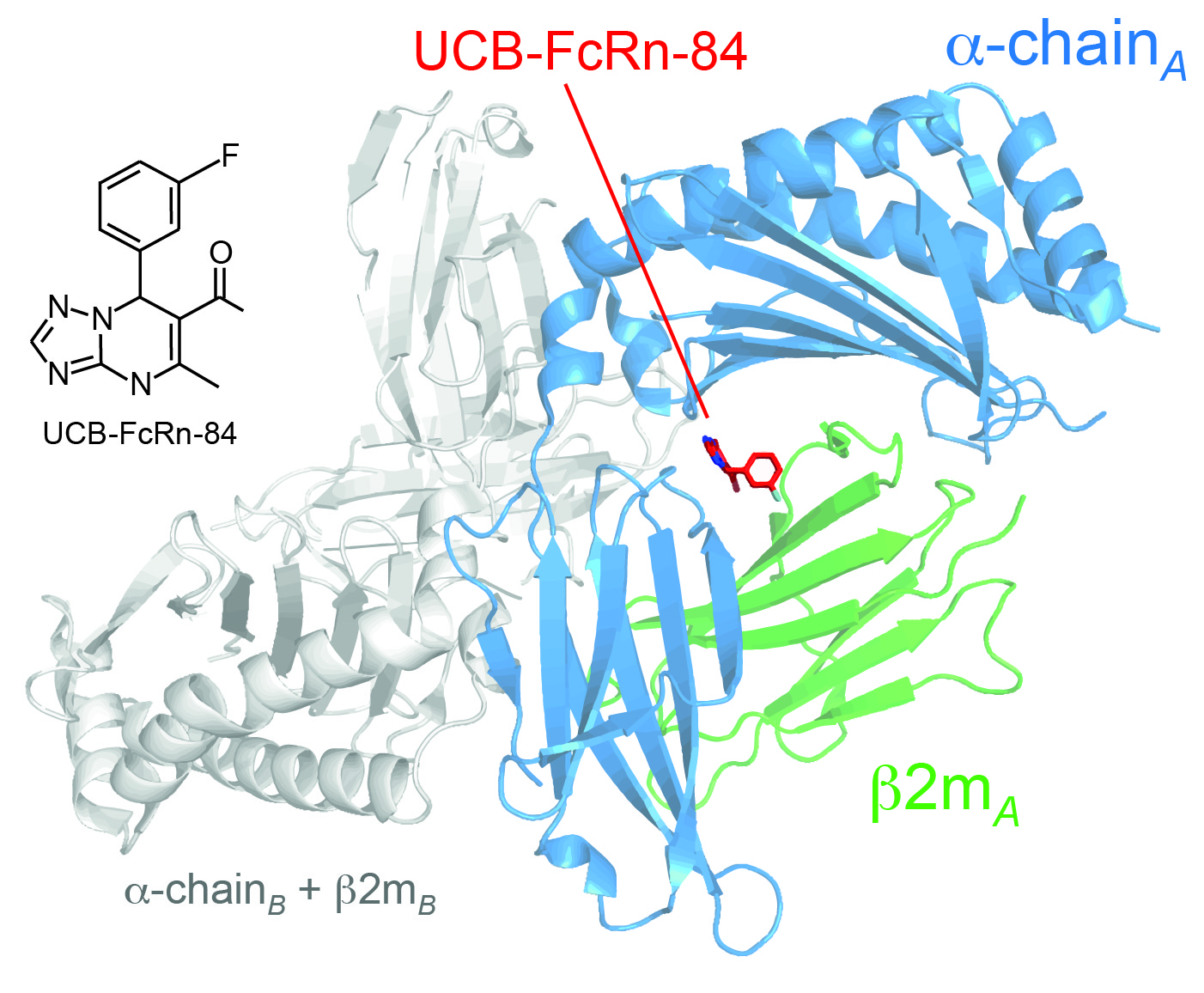

Supplement: S4 Fig — The compound binds at the interface of β2m (green) and the α-chain (blue). Also in this crystal structure, a second heterodimer can be found in the asymmetric unit (grey). β2m, β2-microglobulin; FcRn, neonatal Fc receptor; FcRnECD, extracellular domain of the neonatal Fc receptor. (JPG) [file pbio.2006192.s004.jpg]

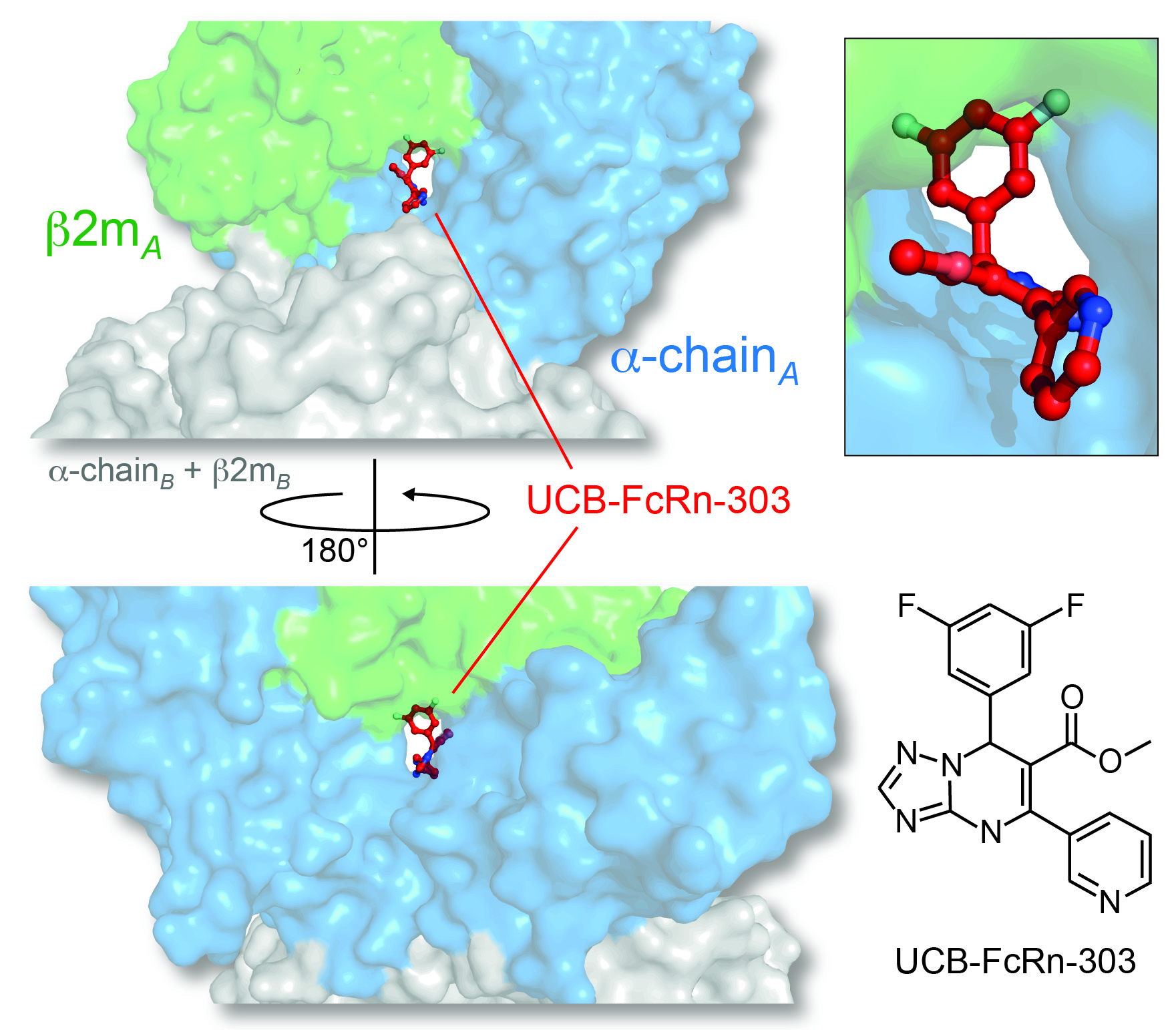

Supplement: S5 Fig — The compound occupies the same binding pocket as UCB-FcRn-84 at the interface of β2m (green) and the α-chain (blue). The binding region is a tunnel-like cavity extending through the protein. Again, a second heterodimer is found in the crystal structure (depicted in grey). β2m, β2-microglobulin; FcRn, neonatal Fc receptor; FcRnECD, extracellular domain of the neonatal Fc receptor. (JPG) [file pbio.2006192.s005.jpg]

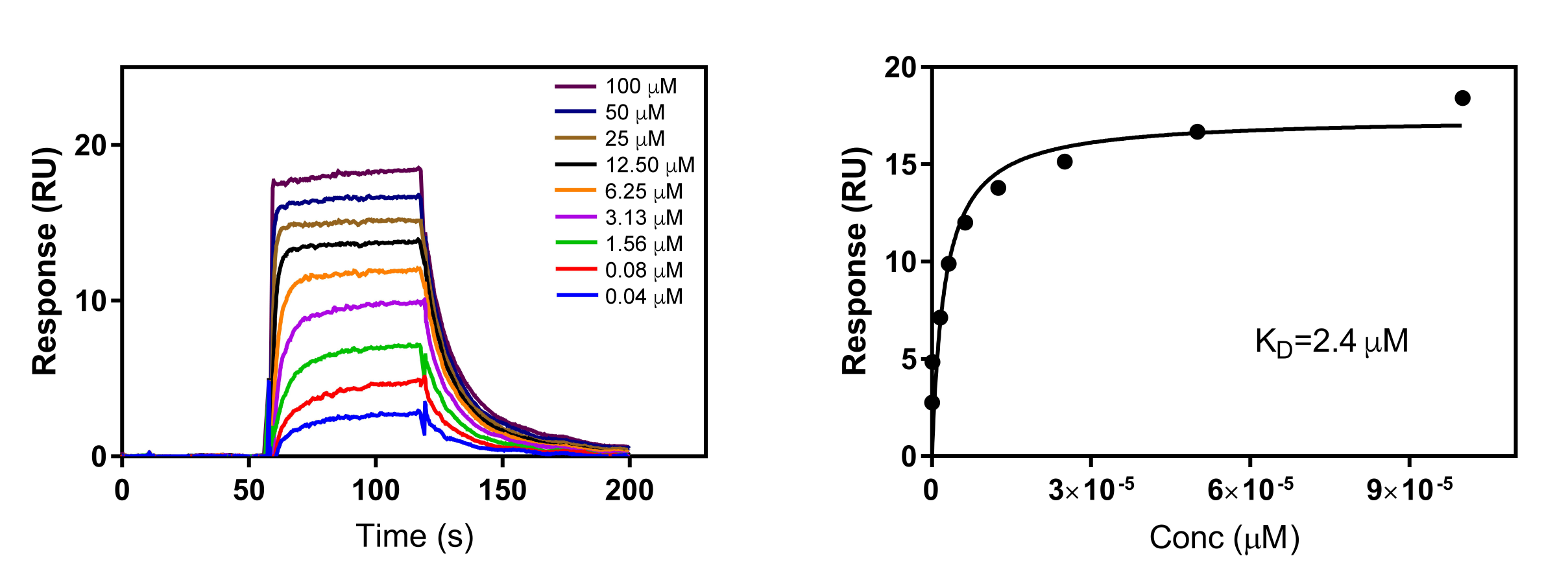

Supplement: S6 Fig — The numerical values can be found in S1 Data. FcRn, neonatal Fc receptor; FcRnECD, extracellular domain of the neonatal Fc receptor; SPR, Surface Plasmon Resonance. (TIF) [file pbio.2006192.s006.tif]

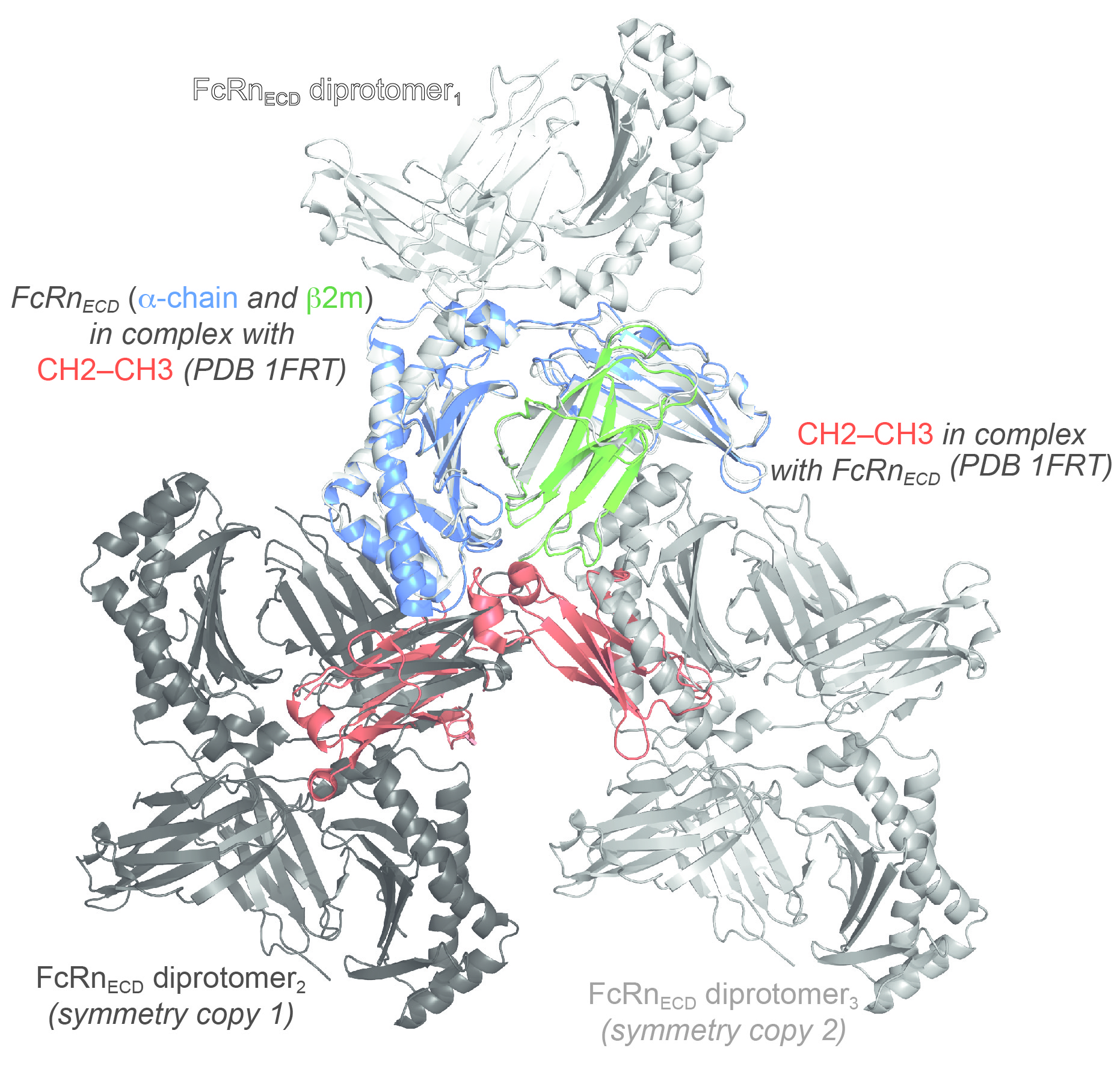

Supplement: S7 Fig — β2m, β2-microglobulin; FcRnECD, extracellular domain of the neonatal Fc receptor; IgG, Immunoglobulin G; PDB, Protein Data Bank. (JPG) [file pbio.2006192.s007.jpg]

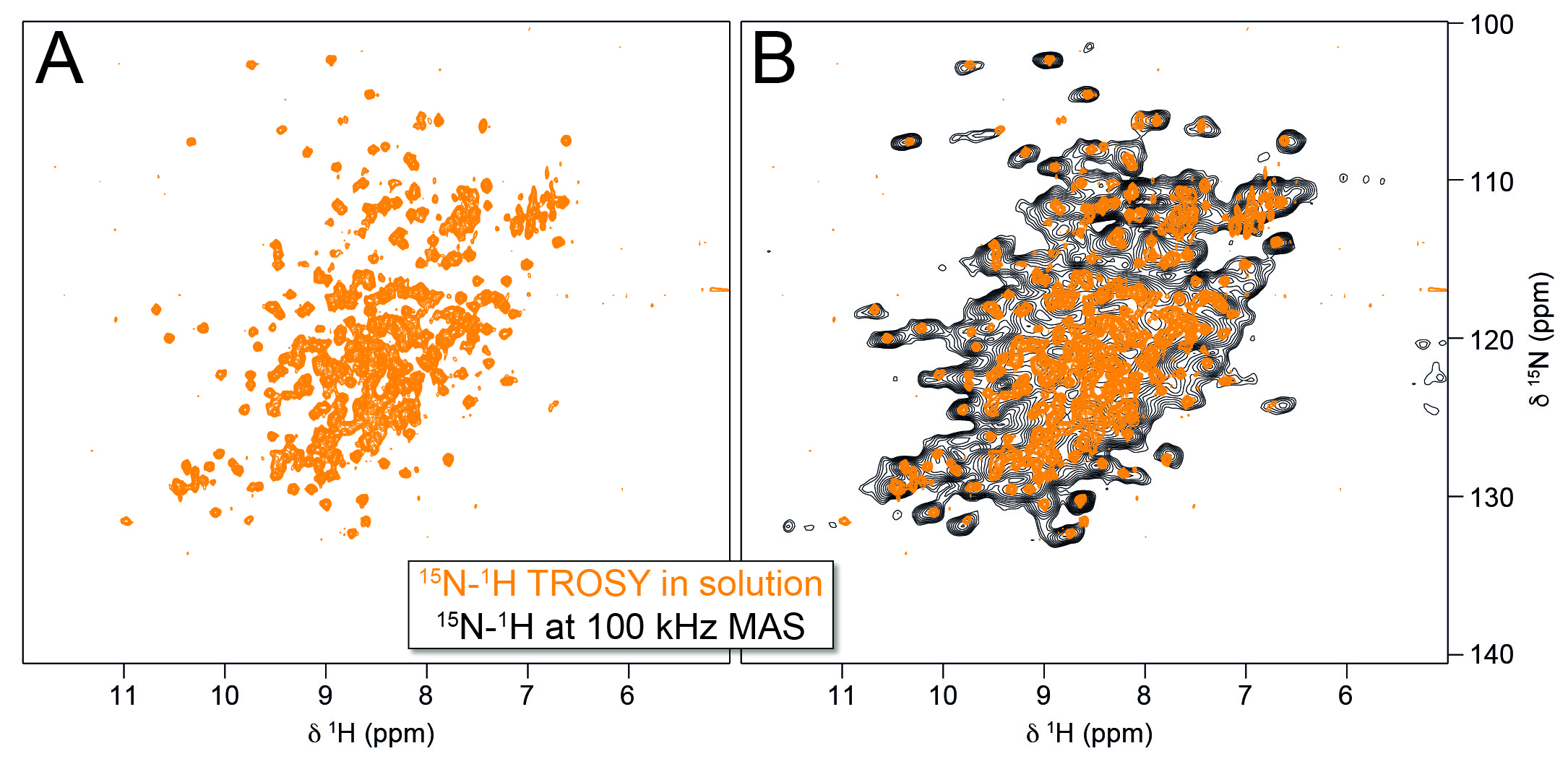

Supplement: S8 Fig — (A) 2D 15N-1H correlation using TROSY of fully protonated [13C,15N]-labeled FcRnECD measured in solution. (B) Overlay of the spectrum shown in (A) (orange) with a 2D 15N-1H spectrum of sedimented fully protonated [13C,15N]-labeled FcRnECD recorded at 100 kHz MAS (black). FcRnECD, extracellular domain of the neonatal Fc receptor; MAS, magic-angle-spinning. (JPG) [file pbio.2006192.s008.jpg]

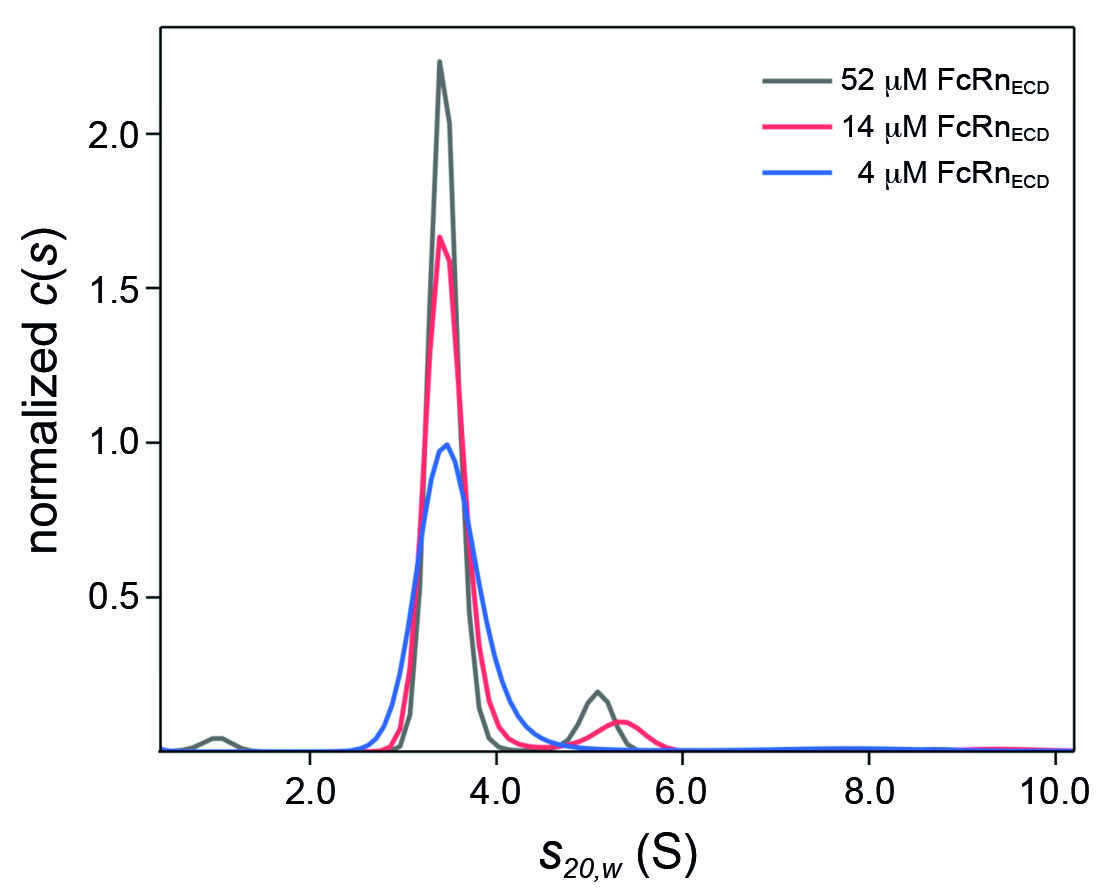

Supplement: S9 Fig — Sedimentation velocity experiments at three different concentrations (52 μM, grey; 14 μM, red; 4 μM, blue) exhibit protein concentration dependent peaks at 3.5 S, 5.1 S, and 5.3 S. FcRnECD, extracellular domain of the neonatal Fc receptor. (JPG) [file pbio.2006192.s009.jpg]

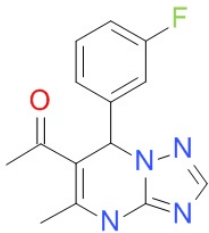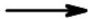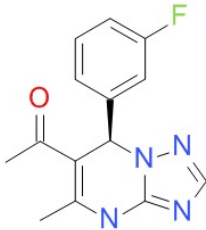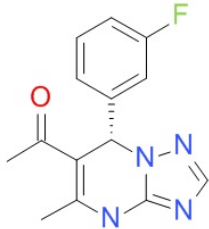

Supplement: S10 Fig — FcRn, neonatal Fc receptor. (PDF) [file pbio.2006192.s010.pdf]

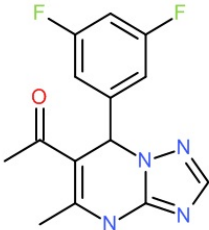

Supplement: S11 Fig — (PDF) [file pbio.2006192.s011.pdf]

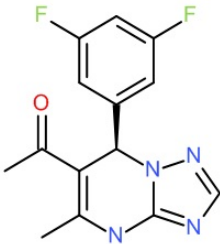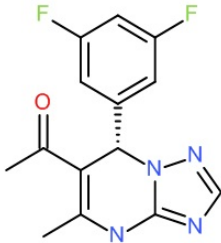

Supplement: S12 Fig — (PDF) [file pbio.2006192.s012.pdf]

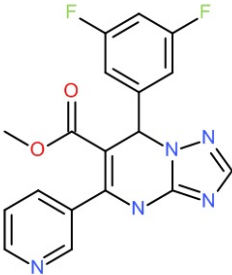

Supplement: S13 Fig — FcRn, neonatal Fc receptor. (PDF) [file pbio.2006192.s013.pdf]
