# Supplementary material for: Insight into small molecule binding to the neonatal Fc receptor by X-ray crystallography and 100 kHz magic-angle-spinning NMR
Source: PLoS Biol. 2018 May 21;16(5):e2006192. doi: 10.1371/journal.pbio.2006192 (PMC5983862; doi:10.1371/journal.pbio.2006192)
Supplement: S1 Table — They are compared to the corresponding chemical-shifts (Beerbaum and colleagues) of [2H,13C,15N]-labeled β2m in MHC1 complexes measured in solution-state NMR [63]. Amino acids of the α-chain are depicted in blue, β2m residues in green. β2m, β2-microglobulin; FcRnECD, extracellular domain of the neonatal Fc receptor; MAS, magic-angle-spinning. (PDF) [file pbio.2006192.s016.pdf]

	^{1}H		^{15}N		$^{13}C\alpha$		$^{13}C\beta$	
	$FcRn_{ECD}$	Beerbaum et al.*	$FcRn_{ECD}$	Beerbaum et al.*	$FcRn_{ECD}$	Beerbaum et al.*	$FcRn_{ECD}$	Beerbaum et al.*
**Arg3**	9.56	9.48	122.24	123.08	55.14	55.84	34.45	34.56
**Pro5**					62.56	62.95		
**Lys6**	9.38	9.29	124.58	124.38	55.82	56.15	33.30	32.87
**Ile7**	8.51	8.49	124.54	124.58	61.47	61.57		42.06
**Ser11**	9.49	9.45	117.98	116.88	56.68	56.44	66.72	65.53
**Arg12**	8.93	8.95	122.56	123.35	60.60	59.51	34.35	33.40
**His13**	8.24	8.40	113.63	114.85	51.36	52.73		30.62
**Ala15**	9.20	9.13	127.28	127.15	53.57	53.27	19.57	19.98
**Glu16**	9.02	9.00	125.30	124.57	54.74	55.31	32.44	32.81
**Asn17**	8.86	8.87	123.30	123.29	54.37	54.68	37.07	37.35
**Gly18**	8.82	8.93	109.15	109.19	45.48	45.57		
**Lys19**	7.96	7.95	121.44	121.48	54.09	54.60	33.54	33.98
**Ser20**	8.53	8.46	120.56	119.95	59.51	59.87	63.14	63.31
**Asn21**	8.97	9.01	127.05	127.03	50.68	51.39	42.14	42.18
**Phe22**	10.45	10.48	119.97	119.81	57.62	58.04	43.93	43.65
**Leu23**	8.98	9.09	125.95	127.21	53.26	53.32	41.66	41.75
**Asn24**	8.26	8.21	121.45	121.73	51.29	51.81	41.37	41.73
**Cys25**	9.68	9.72	120.46	120.96	53.76	54.09	41.69	41.69
**Tyr26**	9.59	9.76	129.31	129.74	55.16	56.38	41.09	41.38
**Val27**	8.93	8.96	130.58	129.07	59.74	60.20	33.18	33.78
**Ser28**	9.13	9.03	118.43	117.71	57.36	57.32	67.70	67.78
**Gly29**	8.32	8.21	108.09	108.20	46.25	46.75		
**Ser33**					61.83			
**Asp34**	7.45	7.28	119.89	120.20	54.91	55.05	40.57	40.58
**Ile35**	8.08	8.05	124.40	123.75	60.64	61.01	41.24	41.02
**Glu36**	8.07	8.10	126.15	125.67	54.97	55.08	32.39	32.76
**Asp38**	8.90	8.94	122.40	122.85	52.57	52.86	46.09	46.30
**Leu39**	9.25	9.19	121.36	121.28	53.77	53.94	44.07	43.97
**Leu40**	9.01	9.06	120.26	120.31	52.92	53.03	45.83	45.85
**Lys41**	8.89	8.89	121.02	121.06	54.13	54.62	34.18	34.41
**Asn42**	9.85	9.84	128.40	128.27	54.51	54.62	37.26	37.38
**Gly43**	8.92	8.92	102.37	102.47	45.37	45.49		
**Glu44**	7.86	7.85	120.79	120.88	54.23	54.65	31.77	32.10
**Arg45**	8.83	8.77	124.58	124.48	57.92	58.04	30.50	30.70
**Ile46**	8.86	8.91	128.71	128.71	61.90	62.15		39.02
**Glu47**	8.59	8.57	126.58	126.43	58.28	58.76	30.74	30.71
**Lys48**	8.10	8.09	119.77	119.73	54.96	56.01		31.00
**Val49**	7.90	7.94	122.35	121.91	55.75	60.98	36.43	34.87
**Glu50**	8.42	8.53	124.54	125.01	54.17	54.71	33.17	32.62
**His51**	8.08	8.15	110.72	112.23	53.67	54.00	30.36	30.78
**Ser52**	9.22	9.19	117.59	116.83	57.69	57.81	66.74	65.91
**Tyr63**		7.63		110.03	57.78	56.40		39.91
**Leu64**	9.23	9.27	120.50	120.10	54.88	55.76		47.20

	^{1}H		^{15}N		$^{13}C\alpha$		$^{13}C\beta$	
	$FcRn_{ECD}$	Beerbaum et al.*	$FcRn_{ECD}$	Beerbaum et al.*	$FcRn_{ECD}$	Beerbaum et al.*	$FcRn_{ECD}$	Beerbaum et al.*
**Leu65**		8.27		122.45	55.48	54.14		46.93
**Tyr66**	9.17	9.20	127.18	127.29	58.02	56.95	42.14	42.26
**Tyr67**	8.91	9.04	116.18	116.75	55.99	56.11	40.95	41.39
**Thr68**	8.35	8.39	111.77	111.81	59.96	60.30	70.33	70.37
**Glu69**	8.60	8.60	130.26	130.19	56.82	56.72	29.83	30.10
**Phe70**	8.81	8.83	126.02	125.79	55.32	55.52	41.35	41.17
**Thr71**	8.47	8.33	117.52	117.28	58.92	58.74	70.18	70.34
**Pro72**					63.15	63.16		
**Thr73**	8.03	8.16	110.49	110.60	60.28	60.37	73.11	73.08
**Glu74**	9.14	9.19	119.15	118.76	58.94	59.09	29.86	30.13
**Lys75**	7.88	7.90	113.75	113.90	56.49	56.38	33.99	34.16
**Asp76**	7.12	7.22	117.95	118.14	55.37	55.58	43.44	43.73
**Glu77**	8.64	8.71	122.68	122.77	54.77	55.18	32.70	33.01
**Tyr78**	9.49	9.56	123.17	123.94	56.84	56.94	43.39	43.28
**Ala79**	8.89	8.87	121.40	121.22	50.97	51.24	23.93	24.40
**Cys80**	9.18	9.20	120.13	120.05	53.02	53.08	43.46	43.63
**Arg81**	9.45	9.51	128.39	128.64	53.83	54.18	34.14	33.91
**Val82**	9.11	9.13	128.30	127.79	60.27	60.56	36.27	35.99
**Asn83**	9.09	9.12	123.10	123.59	51.00	51.36	41.69	41.72
**His84**	7.83	7.82	122.40	122.96	56.84	57.16		36.25
**Val85**	8.23	8.13	126.03	125.15	64.59	64.56	30.99	31.32
**Thr86**	7.52	7.59	110.75	110.74	63.34	63.46	69.90	70.08
**Leu87**	8.02	8.14	123.24	123.29	53.48	53.61		42.95
**Lys91**	8.87	8.84	125.22	125.58	54.67	55.43	35.41	35.40
**Ile92**	8.59	8.60	124.73	125.48	60.51	60.64	39.25	39.12
**Val93**	9.06	9.16	129.45	129.90	61.52	61.70	34.45	34.12
**Lys94**	8.88	8.89	127.70	127.88	56.47	56.61	33.52	33.54
**Trp95**	8.79	8.79	122.20	122.55	57.03	57.08	28.49	28.44
**Asp96**	8.66	8.56	132.16	131.70	52.74	53.07		41.98
**Asp98**	8.31	8.30	121.07	120.38	55.25	54.83		41.38

	^{1}H		^{15}N		$^{13}C\alpha$		$^{13}C\beta$	
	$FcRn_{ECD}$	Beerbaum et al.*	$FcRn_{ECD}$	Beerbaum et al.*	$FcRn_{ECD}$	Beerbaum et al.*	$FcRn_{ECD}$	Beerbaum et al.*
**Pro32**		–		–	64.16	–		–
**Gln33**	7.31	–	117.52	–	58.37	–	28.06	–
**Gln34**	7.59	–	115.00	–	55.91	–	30.37	–
**Tyr35**	7.33	–	121.73	–	54.54	–	37.55	–
**Leu36**	9.96	–	127.22	–	56.49	–	44.95	–
**Ser37**	8.13	–	119.18	–	55.36	–	62.56	–
**Tyr38**	6.51	–	111.44	–	58.68	–	37.11	–
**Asn39**	7.37	–	116.41	–	53.30	–	39.64	–
**Ser40**	9.09	–	119.41	–	60.52	–	63.30	–
**Leu41**	8.80	–	122.35	–	52.64	–	46.11	–
**Pro47**		–		–	61.99	–		–
**Cys48**	9.71	–	131.78	–	53.17	–	42.07	–
**Gly49**	10.28	–	107.51	–	45.58	–		–
**Ala50**	7.86	–	121.24	–	54.20	–	20.06	–
**Trp51**	8.18	–	123.01	–	57.97	–	31.97	–
**Val52**	9.62	–	119.48	–	60.33	–	37.90	–
**Trp53**	7.51	–	117.27	–	53.12	–	29.73	–
**Glu54**	7.87	–	112.12	–	55.52	–	25.88	–
**Gly125**		–		–	45.20	–		–
**Thr126**	8.05	–	112.03	–	59.17	–	69.39	–
**Pro228**		–		–	61.44	–		–
**Asn229**	8.91	–	126.05	–	55.06	–	35.26	–
**Ser230**	8.28	–	117.31	–	59.52	–	63.61	–
**Asp231**	9.40	–	127.73	–	57.21	–	42.33	–
**Gly232**	8.48	–	104.53	–	46.22	–		–
