# Supplementary material for: Insight into small molecule binding to the neonatal Fc receptor by X-ray crystallography and 100 kHz magic-angle-spinning NMR
Source: PLoS Biol. 2018 May 21;16(5):e2006192. doi: 10.1371/journal.pbio.2006192 (PMC5983862; doi:10.1371/journal.pbio.2006192)
Supplement: S2 Table — (PDF) [file pbio.2006192.s017.pdf]

	$FcRn_{ECD}$	$FcRn_{ECD}$–UCB-FcRn-84	$FcRn_{ECD}$–UCB-FcRn-303
**Data Collection**			
Space Group	$P2_1$	$P2_1$	$P2_1$
Cell dimensions			
a, b, c (Å)	42.10, 76.25, 140,15	42.27, 76.29, 138.06	41.77, 76.29, 138.06
α, β, γ (°)	90.000, 93.755, 90.000	90.000, 90.014, 90.000	90.000, 93.561, 90.000
Resolution (Å)	2.00 (2.05-2.00)	1.85 (1.90-1.85)	2.00 (2.05-2.00)
R_{merge} (%)	0.076 (0.545)	0.070 (0.559)	0.071 (0.517)
$I/\sigma(I)$	10.91 (2.39)	12.52 (3.22)	13.96 (2.51)
Completeness (%)	98.8 (98.9)	98.4 (98.0)	99.8 (99.9)
Redundancy	3.00 (3.00)	3.72 (3.77)	3.79 (3.81)
**Refinement**			
Resolution (Å)	2.00	1.85	2.00
No. reflections	59,210	73,773	59,144
R_{work} / R_{free} overall	17.4 / 21.0	17.2 / 21.3	18.9 / 22.0
No. atoms			
Protein	5762	5718	5583
Ligand	18	59	111
Water	486	448	360
ADP ($Å^2$)			
Protein	35.24	28.59	32.88
Ligand	51.93	24.88	35.29
Water	39.85	36.75	37.42
r.m.s. deviations			
Bond lengths (Å)	0.007	0.007	0.007
Bond angles (°)	0.90	0.95	0.91
