# Supplementary material for: Insight into small molecule binding to the neonatal Fc receptor by X-ray crystallography and 100 kHz magic-angle-spinning NMR
Source: PLoS Biol. 2018 May 21;16(5):e2006192. doi: 10.1371/journal.pbio.2006192 (PMC5983862; doi:10.1371/journal.pbio.2006192)
Supplement: S3 Table — FcRnECD, extracellular domain of the neonatal Fc receptor; MAS, magic-angle-spinning. (PDF) [file pbio.2006192.s018.pdf]

Experiment	(H)CANH	(H)CA(CO)NH	(H)CBCANH	(H)CANH (with UCB-FcRn-303)	(H)NH
MAS [kHz]	90	80	80	100	100
Transfer pathway	H-CA-N-H	H-CA-CO-N-H	H-CB-CA-N-H	H-CA-N-H	H-N-H
Transfer 1	H-CA	H-CA	H-CB	H-CA	H-N
Type of transfer	ZQ(n=1) CP	ZQ(n=1) CP	ZQ(n=1) CP	DQ(n=1) CP	DQ(n=1) CP
RF-field ^{1}H [kHz]	125	115	103	75	93
RF-field ^{15}N [kHz]	-	-	-	-	17
RF-field ^{13}C [kHz]	33	34	23	25	-
Shape (channel)	45-55 tangent (^{1}H)	45-55 tangent (^{1}H)	45-55 tangent (^{1}H)	45-55 tangent (^{1}H)	40-60 tangent (^{1}H)
Carrier ^{13}C [ppm]	55	55	17	55	-
Contact time [ms]	1.5	0.75	0.45	0.75	1
Transfer 2	CA-N	CA-CO	CB-CA	CA-N	N-H
Type of transfer	DQ(n=1) CP	DREAM	DREAM	DQ(n=1) CP	DQ(n=1) CP
RF-field ^{1}H [kHz]	-	-	-	-	93
RF-field ^{15}N [kHz]	35	-	-	35	17
RF-field ^{13}C [kHz]	65	41	41	65	-
Shape (channel)	47-53 tangent (^{13}C)	55-45 tangent (^{13}C)	40-60 tangent (^{13}C)	47-53 tangent (^{13}C)	40-60 tangent (^{1}H)
Carrier ^{13}C [ppm]	55	143.4	35	55	-
Contact time [ms]	10	7.5	4	9	1
Transfer 3	N-H	CO-N	CO-N	N-H	
Type of transfer	DQ(n=1) CP	DQ(n=1) CP	DQ(n=1) CP	DQ(n=1) CP	
RF-field ^{1}H [kHz]	93	-	-	81	
RF-field ^{15}N [kHz]	17	35	28	22	
RF-field ^{13}C [kHz]	-	65	48	-	
Shape (channel)	60-40 tangent (^{1}H)	47-53 tangent (^{13}C)	47-53 tangent (^{13}C)	60-40 tangent (^{1}H)	
Carrier ^{13}C [ppm]	-	175	175	-	
Contact time [ms]	1.0	10	10	1.0	
Transfer 4		N-H	N-H		
Type of transfer		DQ(n=1) CP	DQ(n=1) CP		
RF-field ^{1}H [kHz]		93	93		
RF-field ^{15}N [kHz]		17	17		
RF-field ^{13}C [kHz]		-	-		
Shape (channel)		60-40 tangent	60-40 tangent (^{1}H)		
Carrier ^{13}C [ppm]		-	-		
Contact time [ms]		1.0	1.0		
^{1}H pulses [kHz]	150	150	150	150	150
^{13}C pulses [kHz]	100	100	100	100	100
^{15}N pulses [kHz]	62.5	62.5	62.5	62.5	62.5
T1 increments	5500	5500	5500	5500	5500
Sweep widths (t1) [kHz]	46.68	46.68	46.68	46.68	46.68
Max acq. time (t1) [ms]	70	70	70	70	70
T2 increments	60	70	70	56	310
Sweep width (t2) [kHz]	39	37	40	40	110
Max. acq. times (t2) [ms]	8.8	10.9	10.1	8.1	16.4
T3 increments	50	50	200	50	
Sweep width (t3) [kHz]	43	32	80	44	
Max acq. time (t3) [ms]	2.7	3.6	2.3	2.6	
^{1}H decoupling	10 kHz fslp-TPPM	10 kHz fslp-TPPM	10 kHz fslp-TPPM	10 kHz fslp-TPPM	10 kHz fslp-TPPM
Interscan delay [s]	1.0	1.0	1.0	1.0	1.0
Number of scans	128	184	64	56	32
Total measurement time	~ 4 days 19 hours 30 min	~ 8 days 2 hours 51 min	~ 11 days 6 hours 9 min	~ 1 day 23 hours 4 min	~ 2 hours 57 min
