# Supplementary material for: Insight into small molecule binding to the neonatal Fc receptor by X-ray crystallography and 100 kHz magic-angle-spinning NMR
Source: PLoS Biol. 2018 May 21;16(5):e2006192. doi: 10.1371/journal.pbio.2006192 (PMC5983862; doi:10.1371/journal.pbio.2006192)
Supplement: S1 Text — FcRnECD, extracellular domain of the neonatal Fc receptor. (PDF) [file pbio.2006192.s019.pdf]

Ligandability assessments on $FcRn_{ECD}$

In-silico ligandability assessments using SiteMap were performed on $FcRn_{ECD}$ using pH 3 and pH 8.5 crystal structures, in addition to a frame from a molecular dynamics simulation to model a neutral pH crystal structure [1,2].

SiteMap was used to identify binding sites with a propensity for binding small molecules (SiteScore) as well as the druggability score (DScore). At least three distinct sites were observed in each of the three conformations. Site A was predicted to be the most druggable using DScore with the highest potential for binding small molecules calculated by SiteScore. In the pH 7.2 computational model, Site A is merged with Site B. Site D is the next highest ranked site although it is not observed in the pH 3 crystallographic conformation. Although sites C and E are also observed, these are low scoring sites with a weak propensity for ligand binding.

1. Halgren T. Identifying and Characterizing Binding Sites and Assessing Druggability. J Chem Inf Model. 2009;49: 377–389.
2. Halgren T. New Method for Fast and Accurate Binding-site Identification and Analysis. Chem Bio & Drug Design. 2007;69: 146–148.
