# Supplementary material for: Insight into small molecule binding to the neonatal Fc receptor by X-ray crystallography and 100 kHz magic-angle-spinning NMR
Source: PLoS Biol. 2018 May 21;16(5):e2006192. doi: 10.1371/journal.pbio.2006192 (PMC5983862; doi:10.1371/journal.pbio.2006192)
Supplement: S2 Text — FcRn, neonatal Fc receptor. (PDF) [file pbio.2006192.s020.pdf]

UCB-FcRn-303 binds in a tunnel-like cavity with low μM affinity

Both UCB-FcRn-84 and UCB-FcRn-303 bind in the same pocket at the interface of the α-chain and β2m (S4 Fig and S5 Fig). This region of the protein is a tunnel-like hollow space extending through the whole protein (S5 Fig). The compound UCB-FcRn-303 selected for further characterization by NMR using fast MAS shows an affinity of 2.4 μM as measured by SPR (S6 Fig). This corresponds to an increase of 35-fold in affinity compared to UCB-FcRn-84.
