# Supplementary material for: Insight into small molecule binding to the neonatal Fc receptor by X-ray crystallography and 100 kHz magic-angle-spinning NMR
Source: PLoS Biol. 2018 May 21;16(5):e2006192. doi: 10.1371/journal.pbio.2006192 (PMC5983862; doi:10.1371/journal.pbio.2006192)
Supplement: S3 Text — FcRnECD, extracellular domain of the neonatal Fc receptor. (PDF) [file pbio.2006192.s021.pdf]

$FcRn_{ECD}$ adopts a similar structure in solution and sedimented samples

To ensure the overall structure of $FcRn_{ECD}$ is very similar in solution and after sedimentation, we recorded a ^{15}N-^{1}H TROSY spectrum of fully protonated [^{13}C,^{15}N]-labeled $FcRn_{ECD}$ (S8 Fig). The comparison with the ^{15}N-^{1}H correlation spectrum acquired at 100 kHz MAS after sedimentation exhibits only a few chemical-shift differences (S8 Fig). Therefore, we conclude the overall constitution of $FcRn_{ECD}$ after sedimentation to be the same as in solution since the spectra look very similar.
