# Supplementary material for: Insight into small molecule binding to the neonatal Fc receptor by X-ray crystallography and 100 kHz magic-angle-spinning NMR
Source: PLoS Biol. 2018 May 21;16(5):e2006192. doi: 10.1371/journal.pbio.2006192 (PMC5983862; doi:10.1371/journal.pbio.2006192)
Supplement: S4 Text — FcRnECD, extracellular domain of the neonatal Fc receptor. (PDF) [file pbio.2006192.s022.pdf]

Analytical ultracentrifugation reveals a small fraction of dimers of heterodimers at higher concentrations of $FcRn_{ECD}$ in solution

To analyze the formation of dimers of heterodimeric $FcRn_{ECD}$ in solution, we performed sedimentation velocity experiments using Analytical Ultracentrifugation at different protein concentrations (S9 Fig). At all three protein concentrations, the $c(s)$ distribution shows a single, slightly broadened peak at a sedimentation coefficient of 3.5 S. The molecular weight estimation of 44.8 kDa (± 5%) for this detected species is close to the size of one heterodimeric $FcRn_{ECD}$ protomer composed of the α-chain and β2m (42 kDa). The frictional coefficient of approximately 1.3 indicates a globular shape of the protein.

In the measurements with 14 μM and 52 μM $FcRn_{ECD}$, a second species is detected at 5.3 S and 5.1 S, respectively. The estimated molecular weight (83.8 kDa (± 5%)) is close to the one of a dimer of $FcRn_{ECD}$ heterodimers (84 kDa). The population of this fraction increases from 7.6% to 9.5% compared to the respective protomer peaks when increasing the $FcRn_{ECD}$ concentration.
