# Supplementary material for: Insight into small molecule binding to the neonatal Fc receptor by X-ray crystallography and 100 kHz magic-angle-spinning NMR
Source: PLoS Biol. 2018 May 21;16(5):e2006192. doi: 10.1371/journal.pbio.2006192 (PMC5983862; doi:10.1371/journal.pbio.2006192)
Supplement: S5 Text — FcRn, neonatal Fc receptor; MAS, magic-angle-spinning. (PDF) [file pbio.2006192.s023.pdf]

Observed chemical-shifts of $FcRn_{ECD}$ in MAS NMR experiments

All assigned chemical-shifts of sedimented fully protonated [^{13}C,^{15}N]-labeled $FcRn_{ECD}$ observed in the MAS NMR experiments are listed in S1 Table, S2 Data and are deposited in the BMRB (accession number 27437). To allow a comparison with β2m chemical-shifts of [^{2}H,^{13}C,^{15}N]-labeled β2m in complex with unlabeled MHC class I (MHC1) measured in solution-state NMR by Beerbaum et al., these are listed as well (labeled as Beerbaum et al.*) [1]. The published solution-state chemical-shifts could be measured, because deuteration of the MHC1/β2m complex was possible. Chemical-shifts of α-chain residues are not reported in the literature.

1. Beerbaum M, Ballaschk M, Erdmann N, Schnick C, Diehl A, Uchanska-Ziegler B, et al. NMR spectroscopy reveals unexpected structural variation at the protein–protein interface in MHC class I molecules. J Biomol NMR. 2013;57: 167–178.
